# Optimization of Liquid Crystalline Mixtures Enantioseparation on Polysaccharide-Based Chiral Stationary Phases by Reversed-Phase Chiral Liquid Chromatography

**DOI:** 10.3390/ijms25126477

**Published:** 2024-06-12

**Authors:** Magdalena Urbańska

**Affiliations:** Institute of Chemistry, Military University of Technology, ul. Sylwestra Kaliskiego 2, 00-908 Warsaw, Poland; magdalena.urbanska@wat.edu.pl; Tel.: +48-261837549

**Keywords:** racemates, enantiomers, liquid crystals, amylose derivative, cellulose derivative, liquid chromatography

## Abstract

Enantioseparation of nineteen liquid crystalline racemic mixtures obtained based on (R,S)-2-octanol was studied in reversed-phase mode on an amylose tris(3-chloro-5-methylphenylcarbamate) (ReproSil Chiral-MIG) and a cellulose tris(3,5-dichlorophenylcarbamate) (ReproSil Chiral-MIC). These polysaccharide-based chiral stationary phase (CSP) columns for High-Performance Liquid Chromatography (HPLC) were highly effective in recognizing isomers of minor structural differences. The mobile phase (MP), which consists of acetonitrile (ACN)/water (H_2_O) at different volume ratios, was used. The mobile phases were pumped at a flow rate of 0.3, 0.5, or 1 mL·min^−1^ with a column temperature of 25 °C, using a UV detector at 254 nm. The order of the elution was also determined. The chromatographic parameters, such as resolution (R_s_), selectivity (α), and the number of theoretical plates, i.e., column efficiency (N), were determined. The polysaccharide-based CSP columns have unique advantages in separation technology, and this study has shown the potential usefulness of the CSP columns in separating liquid crystalline racemic mixtures belonging to the same homologous series.

## 1. Introduction

Racemic mixtures (racemates) contain optically active compounds called enantiomers; a racemic mixture is a 50:50 mixture of two enantiomers. There are right-handed enantiomers, which can rotate the plane of polarized light by a specific angle to the right, and left-handed enantiomers, which can rotate polarized light by the same angle to the left. Racemates show no optical rotation because the (+) rotation from one enantiomer cancels the (−) rotation from the other exactly [1,2,3]. Enantiomers generally have identical physical properties such as melting point, boiling point, infrared absorptions, and nuclear magnetic resonance (NMR) spectra. It is important to realize, however, that while the melting point of one enantiomer will be identical to that of the other, the melting point of a mixture of the two enantiomers may differ [4].

Modern developments in science and technology have led to the chemical synthesis of many racemic mixtures, of which usually only the enantiomer with the desired properties is used, as was the case with thalidomide [5,6,7,8]. Among all chromatographic techniques, liquid chromatography has achieved a particular development, enabling the analysis of samples in a few minutes. Liquid crystalline compounds and mixtures [9,10,11] can also be analyzed using High-Performance Liquid Chromatography (HPLC). This technique is a commonly used analytical method. This is because it can be used for virtually any analyte or class of analytes, regardless of their properties. It allows you to perform analyses that would be difficult or impossible to perform using other types of chromatography, e.g., gas chromatography (GC), which can be used for organic or inorganic analytes, but the sample must be volatile. HPLC can achieve excellent resolution at high speed and sensitivity for separating high molecular weight polar compounds [12,13,14,15,16,17,18,19,20,21].

Chiral stationary phases (CSPs) are used for direct separation of enantiomers. Due to the principle of operation and their structure, the following can be distinguished [22]:

- polysaccharides,
- Pirkle-type phases,
- cyclodextrins,
- crown ethers,
- ligand exchange phases,
- ion-exchange phases,
- protein phases,
- macrocyclic antibiotics.

Polysaccharides are compounds widely found in nature. These are macromolecular compounds composed of simple sugars connected by glycosidic bonds. There are compounds containing several units, such as oligosaccharides, the simplest of which are disaccharides and polysaccharides. Polysaccharides include, among others, starch, cellulose, and glycogen and have an essential feature: they are optically active [23]. For this reason, they are also used as stationary phases in the separation of mixtures of optically active compounds, and the most frequently used are derivatives of amylose and cellulose [17,21,24,25,26,27,28,29,30,31]. Polysaccharide derivatives embedded in silica gel show a sufficiently high recognition of chiral compounds, but unfortunately, they are only compatible with a limited number of solvents used as mobile phases, those that do not dissolve the stationary phase, such as hydrocarbon solvents and alcohols, acetonitrile, and their aqueous solutions. This limitation in the selection of eluent components is a serious disadvantage because the efficiency depends on the selection of components and the composition of the eluent separation of enantiomers. For this reason, it was decided that the chemical immobilization of polysaccharides on solid support would be the only good solution to expand the range of applicability of various types of solvents as components of mobile phases. The first immobilization of polysaccharide derivatives on silica gel was made using diisocyanate and has been checked by the effectiveness of other methods, such as radical polymerization, photoirradiation, polymerization catalyzed by enzymes, and others. This allowed the use of many other solvents, such as chloroform, dichloromethane, and tetrahydrofuran, as mobile phases of HPLC. In many cases, applying these solvents increased the efficiency of separating enantiomers and allowed for obtaining a different selectivity profile for some racemic mixtures [32,33,34].

The separation mechanism of various enantiomers on chiral stationary phases based on polysaccharides depends mainly on chiral recognition during the formation of reversible types of interactions, creating transient complexes between CSP and the enantiomer. This is achieved by incorporating the enantiomer into grooves occurring in the stationary phase polymer [35,36]. As previously reported [37,38], the chiral recognition abilities of cellulose and amylose phenylcarbamates are controlled mainly by nature and location substituents in phenyl groups. Phenylcarbamates containing electron-donating substituents, such as alkyl groups, or electron-withdrawing substituents, such as halogens, show higher chirality recognition than unsubstituted ones. This effect can be justified by the inductive influence of these substituents on the polarity of the carbamate group and, thus, on the interaction between CSP and the racemates. Electron donor substituents increase the electron density on the carbonyl oxygen of carbamate groups while withdrawing-electron substituents increase the acidity of the NH proton from carbamate groups. The chiral recognition of the phenylcarbamate derivatives is also influenced by the position of the substituents.

Liquid crystal (LC) materials can exhibit a variety of phases depending on the molecular structure and whether the molecule is chiral or achiral [39]. Liquid crystal phases occurring in chiral compounds are marked by adding an asterisk. However, many liquid crystal structures are obtained as racemic mixtures [40], which can be separated into enantiomers using chiral columns. The separation of liquid crystalline racemic mixtures is influenced by:

- volume ratio of solvents used,
- chemical structure of the studied racemic mixtures,
- the type of elution,
- the type of chiral selector.

This work aimed to optimize the separation of nineteen liquid crystalline racemic mixtures obtained based on (R,S)-2-octanol into optically active enantiomers using chiral liquid chromatography on two chiral columns: ReproSil Chiral-MIG and ReproSil Chiral-MIC. These were two immobilized columns from Dr. Maisch [41] with different column packing materials. This work analyzed the influence of the structural features of the studied racemates in terms of their enantioseparation, such as the number of methylene groups and the type of substitution of the benzene ring The mixtures were separated using the isocratic and the gradient elution. During the studies, the type of column, volume ratio of solvents, mobile phase flow rate, and the elution type were changed. The elution order of the enantiomers on these two chiral columns was also determined.

The analyzed racemic mesogens differed in the length of the oligomethylene spacer (n = 2, 3, 5, 6, 7) and the substituents of the benzene ring (X_1_, X_2_ = H or F), and these differences in the structure of the analytes and the type of the polysaccharide selector significantly influence chiral recognition through multiple interactions. It is noted that chiral recognition of racemic solutes on polysaccharide CSPs is achieved through various types of bonding within the chiral helical grooves of the chiral selector, in particular through H-bonding, dipole–dipole and π–π interactions, as well as through steric effects.

The research results allow for determining the suitability of polysaccharide stationary phases for separating liquid crystalline racemates and indicating conditions enabling at least baseline separation of the mixtures into enantiomers. Moreover, the research results suggest how to proceed during enantioseparation with structurally similar materials.

## 2. Results and Discussion

### 2.1. Optimization on the ReproSil Chiral-MIG Column

**Figure 1:**
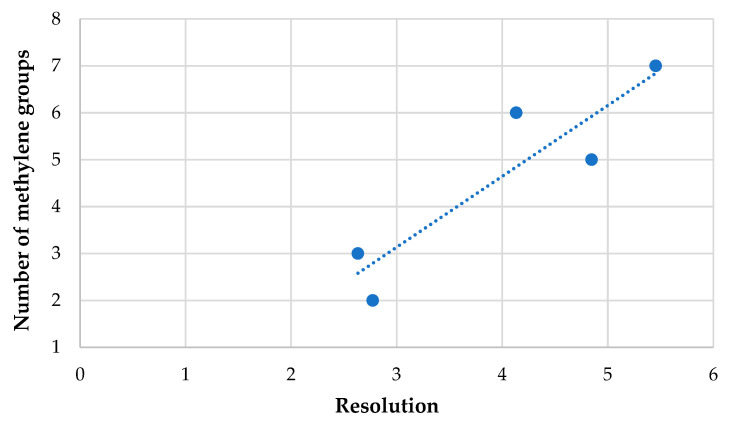
Effect of the oligomethylene spacer length (n) on the resolution values (Rs) on the MIG column for the racemates with (HH) substitution in the acetonitrile–water solvent system in a volume ratio of 95:5.

The analytes were enantiomerically separated using the ACN/H_2_O system without additives. Table 1 summarizes the resolution parameter values for the analyzed racemic mixtures in used elution modes (the highest resolution for a given mixture is underlined, and the lowest is italicized; “-” means no separation). All racemic mixtures were separated in the acetonitrile–water solvent system in a volume ratio of 90:10 and the increasing–decreasing gradient. Four mixtures, 3.(HH) (R,S), 3.(FF) (R,S), 5.(FH) (R,S), and 7.(HF) (R,S), were partially separated in this isocratic elution, and two mixtures, 3.(FF) (R,S) and 7.(HF) (R,S), were partially separated in this gradient elution. The remaining mixtures were baseline-separated. In most cases, the highest resolution values of all five systems are observed in the isocratic elution where there was the most water because materials retained longer achieved a higher resolution in most cases. For mixtures 5.(HH) (R,S), 7.(HF) (R,S), and 7.(HH) (R,S), the highest resolution was obtained in the isocratic elution for 5% water by volume, which may result from long and odd oligomethylene spacers and the substitution of the benzene ring in the ortho position by a fluorine atom, which in turn may influence additional π–π interactions between these analytes and CSP. When comparing analog racemates differing only in the oligomethylene spacer length, it can be seen that increasing the length of the spacer results in higher resolution values, as shown in Figure 1.

In turn, for mixture 3.(FF) (R,S), baseline separation was not achieved in any of the systems; it can be indicated that a short oligomethylene spacer and two fluorine atoms (in the ortho and para positions) hinder the interactions between this analyte and CSP. The materials with two fluorine atoms in the benzene ring also gave the worst results during fast Ultra High-Performance Liquid Chromatography (UHPLC) enantioseparation [42].

The comparison of the resolution of mixtures 3.(FF) (R,S), 5.(HH) (R,S), and 7.HH (R,S), but with another achiral fragment based on (R,S)-2-hexanol [21], is presented in Table 2.

A similar relationship can be observed for these analyzed structures; although the flow rate of the mobile phase was different, the tendency is very similar. If the flow rate was reduced to 0.3 mL·min^−1^ for the mixtures synthesized on the basis (R,S)-2-octanol, the resolution would be quite similar, which means that the substituents of the benzene ring and the number of methylene groups have the greatest impact on the separation process of this group of materials.

**Figure 2:**
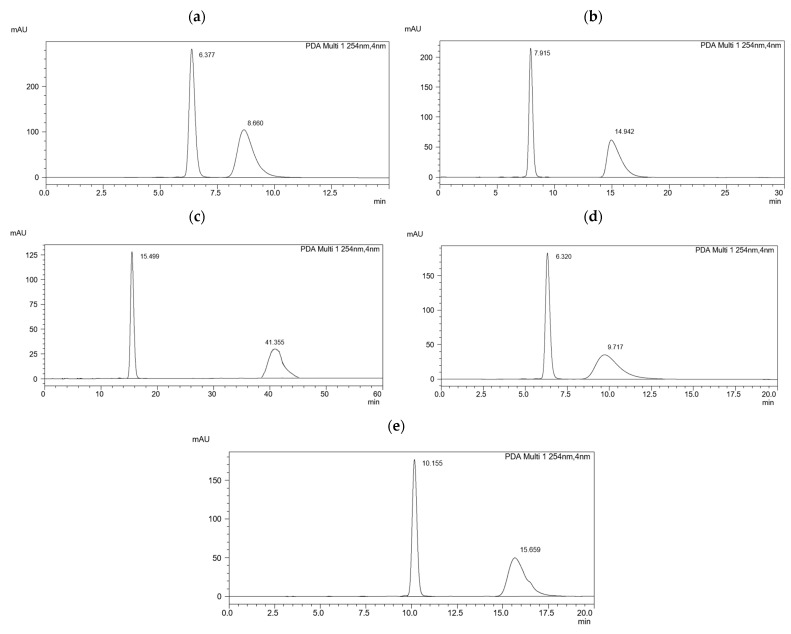
Chromatograms of the racemic mixture 6.(HH) (R,S) obtained on the MIG column in the ACN/H_2_O solvent systems: (**a**) in a volume ratio of 99:1; (**b**) in a volume ratio of 95:5; (**c**) in a volume ratio of 90:10; (**d**) in the decreasing gradient; (**e**) in the increasing–decreasing gradient.

Figure 2a–e shows chromatograms for mixture 6.(HH) (R,S), in which each system is baseline-separated. The highest resolution was obtained in the acetonitrile–water system in a volume ratio of 90:10 and the lowest in a volume ratio of 99:1, which indicates that shorter retention times of analytes result in their weaker interactions with CSPs.

A lower resolution for the decreasing gradient was obtained in the gradient elution. In this type of elution, see Figure 2d,e, significant blurring of the second peak was also observed, which is associated with a deterioration of the efficiency of the chromatographic column.

Table 3 summarizes the retention times (t_R_), selectivity (α), and the number of theoretical plates N (only for baseline-separated mixtures and the first enantiomer—(S)) obtained in the isocratic elution (90:10 *v*/*v*) on the MIG column.

The mixtures with the highest resolution values showed the longest retention times. Retention times were similar for most mixtures; only mixtures with two hydrogen atoms in the benzene ring showed longer retention times. Mixture 5.(HH) (R,S) had the highest ability to produce narrow peaks, while the lowest had mixture 7.(HH) (R,S). The highest selectivity value was achieved for mixture 6.(HH) (R,S) and the lowest for mixture 3.(FF) (R,S).

Figures S1–S12 (in Supplementary Materials) summarize the retention times, selectivity, and the number of theoretical plates obtained for the remaining isocratic and gradient elutions, excluding the system described in this article on the MIG column.

**Figure 3:**
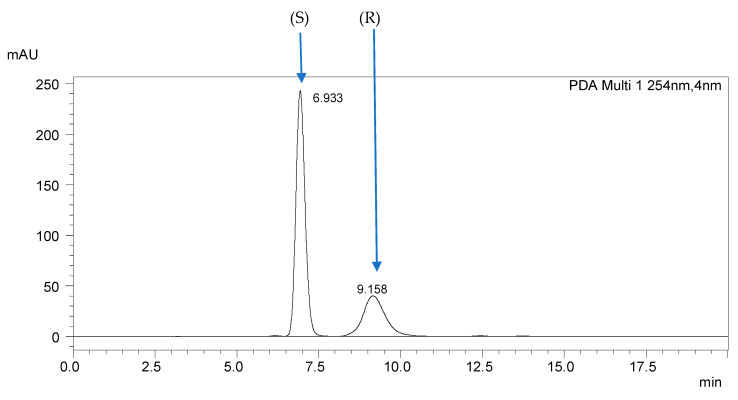
Chromatogram of mixture 6.(FH) (R,S) + (S), obtained on the MIG column in the isocratic elution, ACN/H_2_O (95:5 *v*/*v*), with a flow rate of 1 mL·min^−1^.

Analysis of the racemic mixtures in non-equimolar ratios allowed the determination of the elution order of the enantiomers. The (S) enantiomer was first eluted in all mixtures, as shown in Figure 3, for mixture 6.(FH) (R,S) + (S).

### 2.2. Optimization on the ReproSil Chiral-MIC Column

The second chiral column on which the separation of racemic mixtures was continued was the ReproSil Chiral-MIC column. All previously prepared racemic mixtures were separated using the isocratic and the gradient elution. The same analysis parameters were used for the MIC column (as shown in Tables in Section 3.3), except that the flow rate for the ACN/H_2_O (99:1 *v*/*v*) system was reduced to 0.5 mL min^−1^, and for ACN/H_2_O (95:5 *v*/*v*) and (90:10 *v*/*v*), up to 0.3 mL·min^−1^.

**Figure 4:**
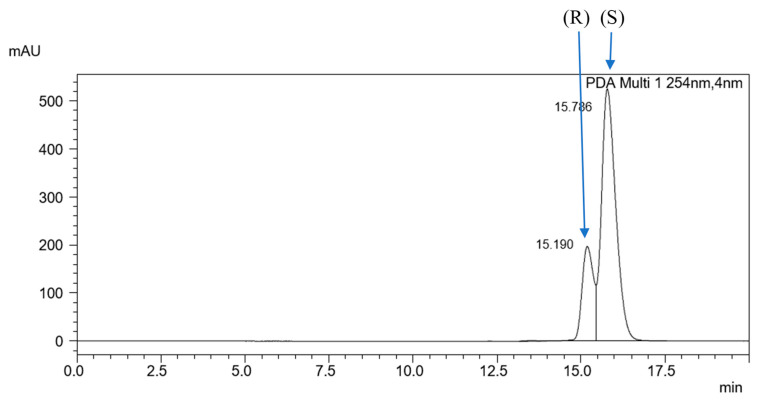
Chromatogram of mixture 6.(HH) (R,S) + (S) obtained on the MIC column in the isocratic elution, ACN/H_2_O (95:5 *v*/*v*), with a flow rate of 0.3 mL·min^−1^.

Analysis of racemic mixtures in non-equimolar ratios allowed the determination of the elution order of the enantiomers. Unlike the ReproSil Chiral-MIG column, the (R) enantiomer was the first to elute during the analysis of the racemic mixtures on the ReproSil Chiral-MIC column (Figure 4).

**Figure 5:**
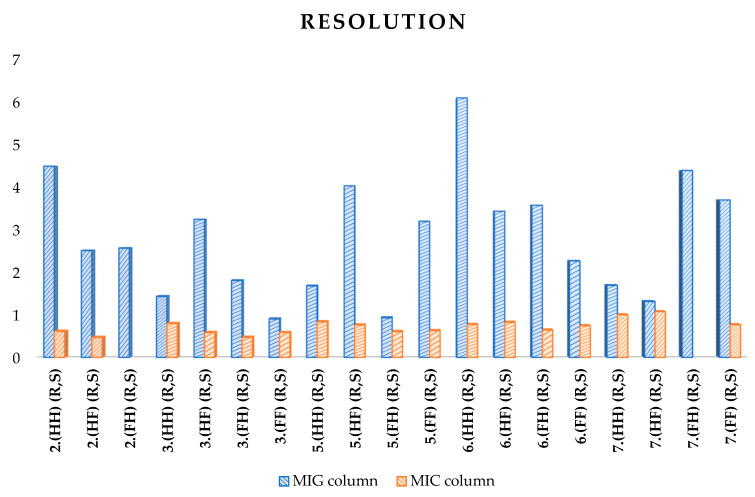
Comparison of the resolution parameter on two columns—ACN/H_2_O (90:10 *v*/*v*).

On the MIC column, none of the mixtures were baseline-separated, regardless of the elution used, and the best results (partial separation of several mixtures) were obtained for the ACN/H_2_O (90:10 *v*/*v*) system. Figure 5 visualizes the comparison of the resolution parameter for both columns and the conditions indicated above.

In each case, the amylose column has a higher resolution; even reducing the flow rate on the cellulose column did not significantly improve the resolution. The highest resolution (over 6) was obtained for mixture 6.(HH) (R,S) on the MIG column.

**Figure 6:**
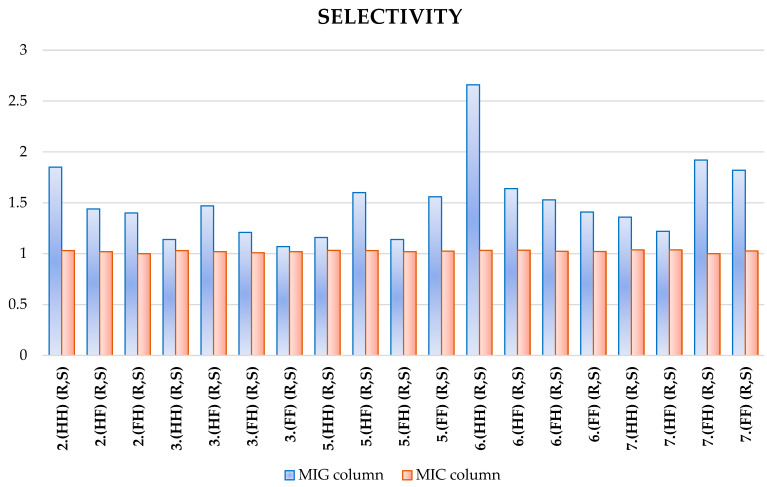
Comparison of the selectivity parameter on two columns.

The selectivity parameter for these two columns was also compared, as shown in Figure 6. On the MIC column, this value exceeds at least one; on the MiG column, it is always higher, and in the case of mixture 6.(HH) (R,S), it exceeds a value of 2.5.

These results indicate that the different structural features of the CSPs, in combination with the mobile phase ACN/H_2_O, ultimately lead to a different stereo-environment of the chiral cavities in the CSPs, resulting in different chiral selectivities.

**Figure 7:**
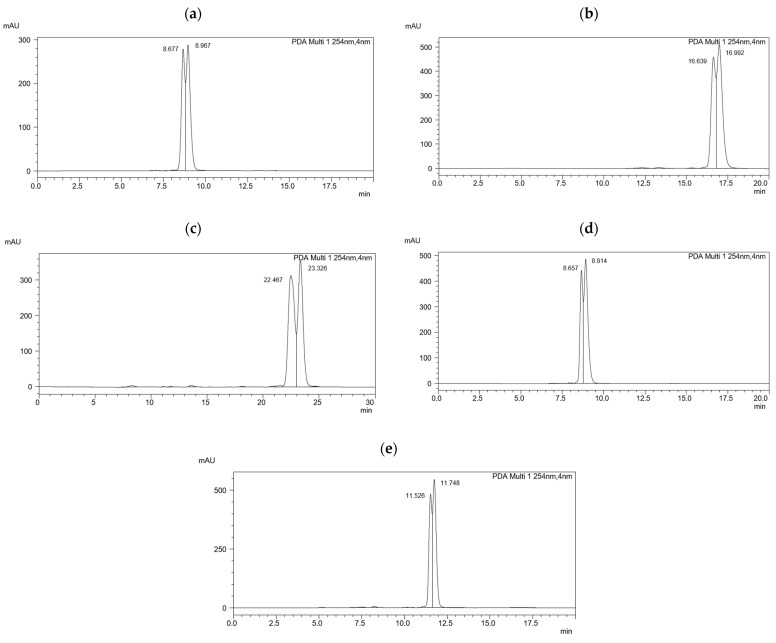
Chromatograms of the racemic mixture 7.(HF) (R,S) obtained on the MIC column in the ACN/H_2_O solvent systems: (**a**) in a volume ratio of 99:1; (**b**) in a volume ratio of 95:5; (**c**) in a volume ratio of 90:10; (**d**) in the decreasing gradient; (**e**) in the increasing–decreasing gradient.

Figure 7a–e shows chromatograms for mixture 7.(HF) (R,S), which separated best in all five systems considered on the MIC column. Similar to the MIG column, the influence of the length of the oligomethylene spacer and fluorination in the ortho position on the separation process is visible. In the isocratic elution, as the concentration of acetonitrile in the solvent system decreased, the retention time of the peaks increased. The highest resolution was obtained in the acetonitrile–water system in a volume ratio of 90:10 and the lowest in a volume ratio of 95:5. In gradient elution, much lower parameters were obtained for the increasing–decreasing gradient, which showed similar parameter values to the isocratic system in a volume ratio of 95:5.

Table 4 summarizes the resolution parameter values for the racemic mixtures in all elution modes obtained on the MIC column (the highest resolution for a given mixture is underlined, and the lowest is italicized; “-” means no separation).

In the Supplementary Materials, Figures S13–S22 show the retention times and selectivity for the MIC column in all elution types for all racemic mixtures. The number of theoretical plates was not counted because baseline separation was not observed for any mixture.

In this column, the resolutions very rarely exceeded a value of one. Non-fluorinated mixtures separated best, but baseline separation was not achieved for any of them.

**Figure 8:**
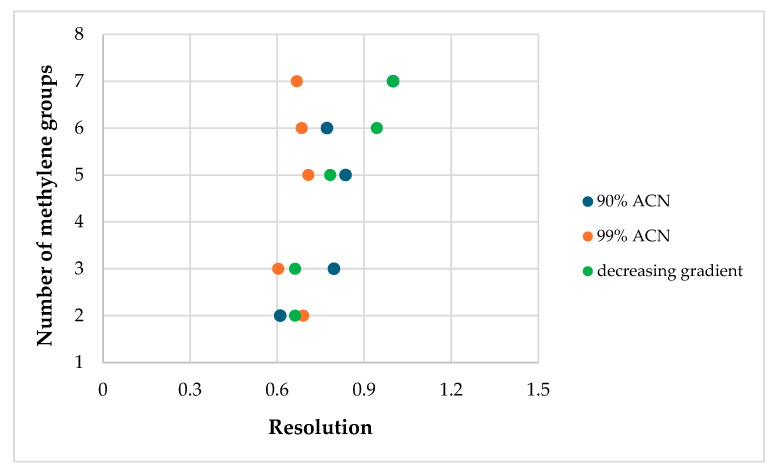
Effect of the oligomethylene spacer length (n) on the resolution values (Rs) on the MIC column for the racemates with (HH) substitution in the acetonitrile–water solvent system in a volume ratio of 90:10 and 99:1 and decreasing gradient.

Mixture 2.(FH) (R,S) was not separated in any system. The mixtures with a shorter oligomethylene spacer (n = 2 and 3) separated much worse in this column (see Figure 8), which was also observed in the case of the MIG column.

Moreover, this column filled with cellulose had two substituents of the phenyl group, and these were chlorine atoms, which is also important because in the group of columns of polysaccharide derivatives of cellulose, there are also those with one substituent in the form of a methyl group, and here different separation results should be expected.

In the case of enantioseparation on the cellulose derivatives, better results are usually obtained in the normal phase system and at elevated temperatures, as in Ref. [16].

## 3. Materials and Methods

### 3.1. Racemic Mixtures

**Figure 9:**
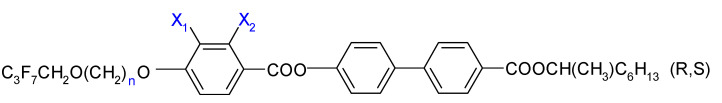
The general formula of the racemic mixtures used in the studies, where n is the length of the oligomethylene spacer (n = 2, 3, 5, 6, 7), and X_1_ and X_2_ are substituents of the benzene ring (fluorine or hydrogen).

The studies concerned nineteen racemic mixtures differing in the oligomethylene spacer’s length and the benzene ring’s substituents (see Figure 9).

Depending on the number of methylene groups and the substitution of the benzene ring, each mixture was given an appropriate acronym (Table 5).

These mixtures were synthesized as shown in Refs. [43,44]. The studied racemates were prepared by treating (R,S) 4′-(1-methylheptyloxycarbonyl)biphenol with a benzoic acid chloride. The efficient preparation of achiral biphenol is described in Ref. [45]. The commercially available (R,S)-2-octanol with a purity of 99.5% was used. The studied racemates are rod-like liquid crystals with smectic phases (SmC_A_ and/or SmC and/or SmA).

### 3.2. Equipment

**Figure 10:**
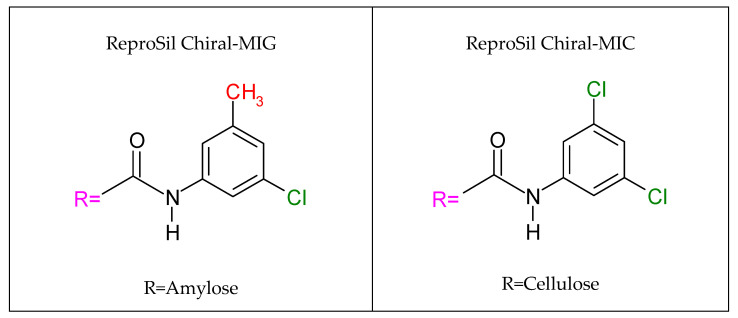
Schematic structure of the chiral stationary phases of the columns used.

Enantioseparation was performed on a Shimadzu LC-20AP HPLC system (Kyoto, Japan), which consists of a binary solvent delivery pump, an autosampler (SIL-10AP), a communications bus module (CBM-20A), a diode array detector (SPD-M20A), and a fraction collector (FRC-10A). Chromatographic data acquisition and analysis were performed using Shimadzu software (Labsolutions, 2010–2017 Shimadzu Corporation). Chiral stationary phases (CSPs) made by covalent immobilization of amylose tris(3-chloro-5-methylphenylcarbamate) on silica gel (ReproSil Chiral-MIG) and cellulose tris(3,5-dichlorophenylcarbamate) on silica gel (ReproSil Chiral-MIC) were used; see Figure 10. The columns had a particle size of 5 μm, dimensions of 250 mm × 4.6 mm i.d, and a pore size of 1000 Å (Dr. Maisch, Ammerbuch-Entringen, Germany).

Based on the polysaccharide’s CSP, three key regions of strongly hanging groups contribute to the retention and selectivity of enantiomers, namely electrophilic amide hydrogen (N–H), nucleophilic carbonyl oxygen (C=O), and π–π electronic cloud [46].

### 3.3. Chromatographic Conditions and Calculations

The analytical method was developed, and different combinations of acetonitrile and water were used as the mobile phase. Elution was performed in the isocratic and gradient modes (Table 6 and Table 7). The sample concentrations were 0.5–0.6 mg·mL^−1^. The samples were dissolved in ACN, which was used as purchased (ACN—POCH S.A.). In addition, ultrapure water was used. Samples of non-equimolar mixtures, (R,S) + (S) or (R,S) + (R), were also prepared to determine the elution order. The injection volume of samples was 10 or 15 μL. The mobile phase flow rate was 0.3, 0.5, or 1 mL·min^−1^. The separation was carried out at 25 °C. The detection wavelength was fixed at 254 nm.

The number of theoretical plates (N), separation factors (α), and resolution factors (Rs) were calculated using the equations presented in Ref. [47]. The most crucial thing in HPLC is to obtain the optimum resolution in the minimum time. A resolution value of 1.5 or greater between two peaks will ensure that the sample components are well (baseline) separated to a degree at which the area or height of each peak may be accurately measured.

The plate number (N) measure the HPLC column’s peak dispersion, reflecting the column performance. Therefore, the more theoretical plates available within the column, the more equilibrations are possible, and the better the quality of the separation (the column with a high number of plates gives narrower, more efficient peaks).

The selectivity parameter α is the ability of a chromatographic system to “chemically” distinguish between sample components. The mixtures that separated and had the largest difference in retention times showed the highest selectivity value. The value of this parameter for mixtures that have not separated equals 1. As the selectivity of the separation depends upon the chemistry of the analyte, mobile, and stationary phases, all of these factors may be altered to change or optimize the selectivity of the HPLC separation.

## 4. Conclusions

This article demonstrates the potential of polysaccharide columns for separating liquid crystalline racemates. As many as seventeen racemic mixtures were baseline-separated in the increasing–decreasing gradient on the Reprosil Chiral-MIG column. The highest resolution for most mixtures was achieved in the isocratic elution in the ACN/H_2_O solvent system in a volume ratio of 90:10, proving that the reversed-phase mode has the potential for analysis of liquid crystals that are usually analyzed in normal mode. Racemates without lateral fluorine substitution are separated the best, and those with double fluorine substitution are the worst. Materials with longer spacers are better separated, and enantioselectivity increases. Regardless of the column used, the longest retention times are observed for non-fluorinated mixtures with (HH) substitution.

None of the racemic mixtures on the ReproSil Chiral-MIC column were baseline-separated, regardless of the elution. The best results were obtained for the isocratic elution, where the water volume ratio in the mobile phase was the highest (seventeen mixtures were partially separated).

The enantiomer elution order depends on the column packing material; in the amylose column, the (S) enantiomer elutes first, while the opposite occurs in the cellulose column.

In summary, better results were obtained on the column packed with amylose and having two phenyl group substituents (-CH_3_ and -Cl), which suggests that the interactions between these analytes and CSPs were the strongest. The results show a U-shaped retention phenomenon. This tendency is observed when the mixture contains an aromatic core in its structure and is analyzed in mobile phases containing large amounts of acetonitrile [48].

The influence of analyte structure on enantioseparation was assessed. Further experiments are planned in the future to investigate the possibility of using other mobile phases and polysaccharide columns for the enantioseparation of these analytes.

## Figures and Tables

**Table 1:** The resolution obtained on the ReproSil Chiral-MIG column in all elution types for the studied racemates.

Resolution					
Acronym of the Racemic Mixtures	ACN/H_2_O 99/1 (*ν*/*ν*)	ACN/H_2_O 95/5 (*ν*/*ν*)	ACN/H_2_O 90/10 (*ν*/*ν*)	Decreasing Gradient	Increasing–Decreasing Gradient
**2.(HH) (R,S)**	1.301	2.773	4.482	*1.131*	2.905
**2.(HF) (R,S)**	-	*1.301*	2.503	-	1.809
**2.(FH) (R,S)**	*0.696*	1.471	2.561	0.933	1.794
**3.(HH) (R,S)**	1.262	2.632	1.428	*1.149*	3.021
**3.(HF) (R,S)**	*0.767*	1.653	3.237	0.822	2.330
**3.(FH) (R,S)**	*0.509*	1.080	1.802	0.554	1.526
**3.(FF) (R,S)**	-	-	0.900	-	*0.630*
**5.(HH) (R,S)**	2.498	4.847	*1.675*	2.120	4.391
**5.(HF) (R,S)**	1.878	3.757	4.021	*1.590*	4.744
**5.(FH) (R,S)**	1.056	1.672	0.928	*0.880*	3.098
**5.(FF) (R,S)**	0.736	1.632	3.188	*0.606*	2.665
**6.(HH) (R,S)**	*1.942*	4.133	6.083	2.123	3.795
**6.(HF) (R,S)**	1.773	3.116	3.425	*1.574*	4.989
**6.(FH) (R,S)**	1.008	1.637	3.565	*0.953*	2.983
**6.(FF) (R,S)**	0.440	1.027	2.260	*0.368*	1.845
**7.(HH) (R,S)**	2.845	5.454	1.689	2.135	*1.536*
**7.(HF) (R,S)**	2.650	5.196	1.310	1.627	*0.969*
**7.(FH) (R,S)**	1.436	2.353	4.382	*1.348*	3.195
**7.(FF) (R,S)**	0.921	2.116	3.690	*0.910*	2.879

**Table 2:** The resolution obtained on the ReproSil Chiral-MIG column for the racemates with different achiral chains.

Resolution, MIG Column		
Acronym of the Racemic Mixtures	ACN/H_2_O 90/10 (*ν*/*ν*), 1 mL·min^−1^	ACN/H_2_O 90/10 (*ν*/*ν*), 0.3 mL·min^−1^
**3.(FF) (R,S) based on (R,S)-2-octanol**	0.900	-
**3.(FF) (R,S) based on (R,S)-2-hexanol**	-	0.890
**5.(HH) (R,S) based on (R,S)-2-octanol**	1.675	-
**5.(HH) (R,S) based on (R,S)-2-hexanol**	-	2.254
**7.(HH) (R,S) based on (R,S)-2-octanol**	1.689	-
**7.(HH) (R,S) based on (R,S)-2-hexanol**	-	2.640

**Table 3:** System suitability parameters of the proposed HPLC method using mobile phase ACN/H_2_O (90:10 by volume).

Acronym of the Racemic Mixtures	t_R_ [min]		α	N_S_
	Enantiomer 1	Enantiomer 2		
**2.(HH) (R,S)**	9.371	17.328	1.849	2200
**2.(HF) (R,S)**	7.627	11.007	1.443	1900
**2.(FH) (R,S)**	8.165	11.431	1.400	1650
**3.(HH) (R,S)**	6.180	7.037	1.138	-
**3.(HF) (R,S)**	7.932	11.665	1.469	2050
**3.(FH) (R,S)**	8.112	9.824	1.211	2150
**3.(FF) (R,S)**	7.204	7.699	1.068	-
**5.(HH) (R,S)**	14.933	17.278	1.157	5600
**5.(HF) (R,S)**	7.000	11.223	1.603	3100
**5.(FH) (R,S)**	5.918	6.754	1.141	-
**5.(FF) (R,S)**	8.548	13.331	1.559	1850
**6.(HH) (R,S)**	15.499	41.355	2.668	950
**6.(HF) (R,S)**	6.159	10.098	1.639	950
**6.(FH) (R,S)**	12.306	18.903	1.536	1700
**6.(FF) (R,S)**	9.583	13.539	1.412	1500
**7.(HH) (R,S)**	7.543	10.288	1.369	600
**7.(HF) (R,S)**	5.970	7.280	1.219	-
**7.(FH) (R,S)**	14.211	27.357	1.925	800
**7.(FF) (R,S)**	11.297	20.524	1.816	2050

**Table 4:** The resolution obtained on the ReproSil Chiral-MIC column in all elution types.

Resolution					
Acronym of the Racemic Mixtures	ACN/H_2_O 99/1 (*ν*/*ν*)	ACN/H_2_O 95/5 (*ν*/*ν*)	ACN/H_2_O 90/10 (*ν*/*ν*)	Decreasing Gradient	Increasing–Decreasing Gradient
**2.(HH) (R,S)**	0.690	-	*0.611*	0.662	0.612
**2.(HF) (R,S)**	0.471	-	0.466	*0.434*	-
**2.(FH) (R,S)**	-	-	-	-	-
**3.(HH) (R,S)**	0.604	0.702	0.796	0.662	*0.502*
**3.(HF) (R,S)**	*0.577*	-	0.582	-	-
**3.(FH) (R,S)**	-	-	0.469	*0.425*	-
**3.(FF) (R,S)**	-	-	0.583	-	-
**5.(HH) (R,S)**	*0.708*	0.965	0.836	0.783	0.736
**5.(HF) (R,S)**	0.602	0.674	0.761	*0.517*	-
**5.(FH) (R,S)**	*0.465*	-	0.604	-	-
**5.(FF) (R,S)**	*0.390*	-	0.628	-	-
**6.(HH) (R,S)**	0.685	1.112	0.772	0.944	*0.597*
**6.(HF) (R,S)**	0.635	-	0.824	0.513	*0.474*
**6.(FH) (R,S)**	0.628	-	0.640	*0.454*	-
**6.(FF) (R,S)**	0.531	*0.500*	0.743	0.592	0.510
**7.(HH) (R,S)**	0.668	0.661	1.000	1.000	*0.549*
**7.(HF) (R,S)**	0.725	*0.588*	1.073	0.734	0.740
**7.(FH) (R,S)**	0.595	*0.433*	-	0.672	0.487
**7.(FF) (R,S)**	*0.482*	0.852	0.768	0.696	-

**Table 5:** The acronyms of the racemic mixtures.

2.(HH) (R,S)	3.(HH) (R,S)	5.(HH) (R,S)	6.(HH) (R,S)	7.(HH) (R,S)
2.(HF) (R,S)	3.(HF) (R,S)	5.(HF) (R,S)	6.(HF) (R,S)	7.(HF) (R,S)
2.(FH) (R,S)	3.(FH) (R,S)	5.(FH) (R,S)	6.(FH) (R,S)	7.(FH) (R,S)
-	3.(FF) (R,S)	5.(FF) (R,S)	6.(FF) (R,S)	7.(FF) (R,S)

**Table 6:** The analysis parameters on the ReproSil Chiral-MIG column in the isocratic elution.

No.	*v*/*v*		Flow Rate [mL·min^−1^]	Injection Volume [µL]
	ACN	H_2_O		
1.	99	1	1	15
2.	95	5	1	15
3.	90	10	1	15

**Table 7:** The analysis parameters on the ReproSil Chiral-MIG column in the gradient elution.

Decreasing Gradient			Increasing–Decreasing Gradient		
Time [min]	ACN/H_2_O (*v*/*v*)	Injection Volume [µL]	Time [min]	ACN/H_2_O (*v*/*v*)	Injection Volume [µL]
0.01	99/1	10	0.01	92/8	10
5	96/4		3	94/6	
10	93/7		6	96/4	
15	90/10		9	98/2	
20	STOP		12	95/5	
-	-		15	92/8	
-	-		20	STOP	

## Data Availability

The data presented in this study are available on request from the corresponding author.

## Supplementary Materials

The following supporting information can be downloaded at https://www.mdpi.com/article/10.3390/ijms25126477/s1.
