# Supplementary material for: Optimization of Liquid Crystalline Mixtures Enantioseparation on Polysaccharide-Based Chiral Stationary Phases by Reversed-Phase Chiral Liquid Chromatography
Source: Int J Mol Sci. 2024 Jun 12;25(12):6477. doi: 10.3390/ijms25126477 (PMC11203475; doi:10.3390/ijms25126477)
Supplement: Supplementary file 1 [file ijms-25-06477-s001.zip › ijms-3028947-supplementary.pdf]

Supplementary materials for the article

Optimization of Liquid Crystalline Mixtures Enantioseparation on Polysaccharide-Based Chiral Stationary Phases by Reversed-Phase Chiral Liquid Chromatography

Magdalena Urbańska

Institute of Chemistry, Military University of Technology, ul. Sylwestra Kaliskiego 2, 00-908 Warsaw, Poland; magdalena.urbanska@wat.edu.pl; Tel.: +48-261837549

RETENTION TIMES, NUMBER OF THEORETICAL PLATES, AND SELECTIVITY OF THE RACEMIC MIXTURES

Figures S1-S22 show the retention times, the number of theoretical plates, and selectivity for both columns.

REPROSIL CHIRAL-MIG COLUMN

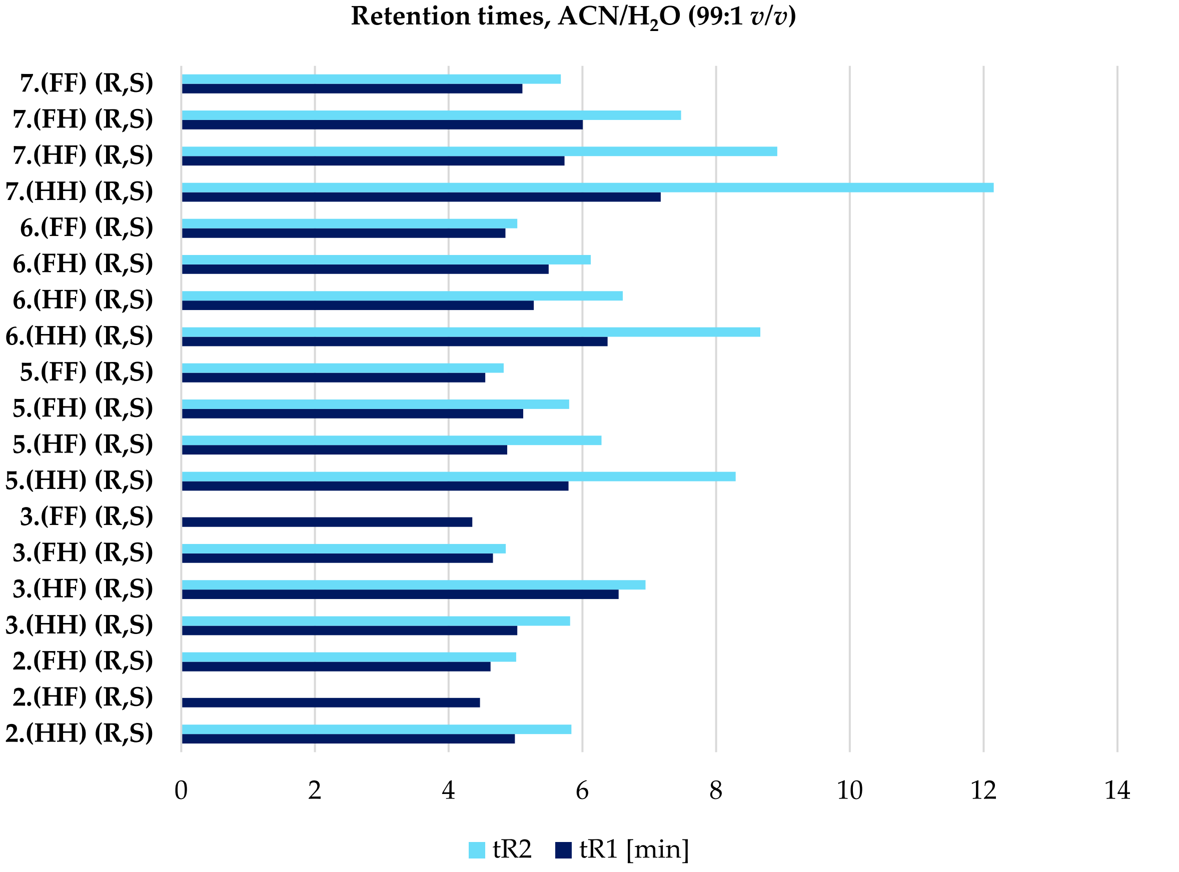

Figure S1. The retention times of the racemic mixtures on the MIG column in the isocratic elution, ACN/H_2O (99:1 *v/v*).

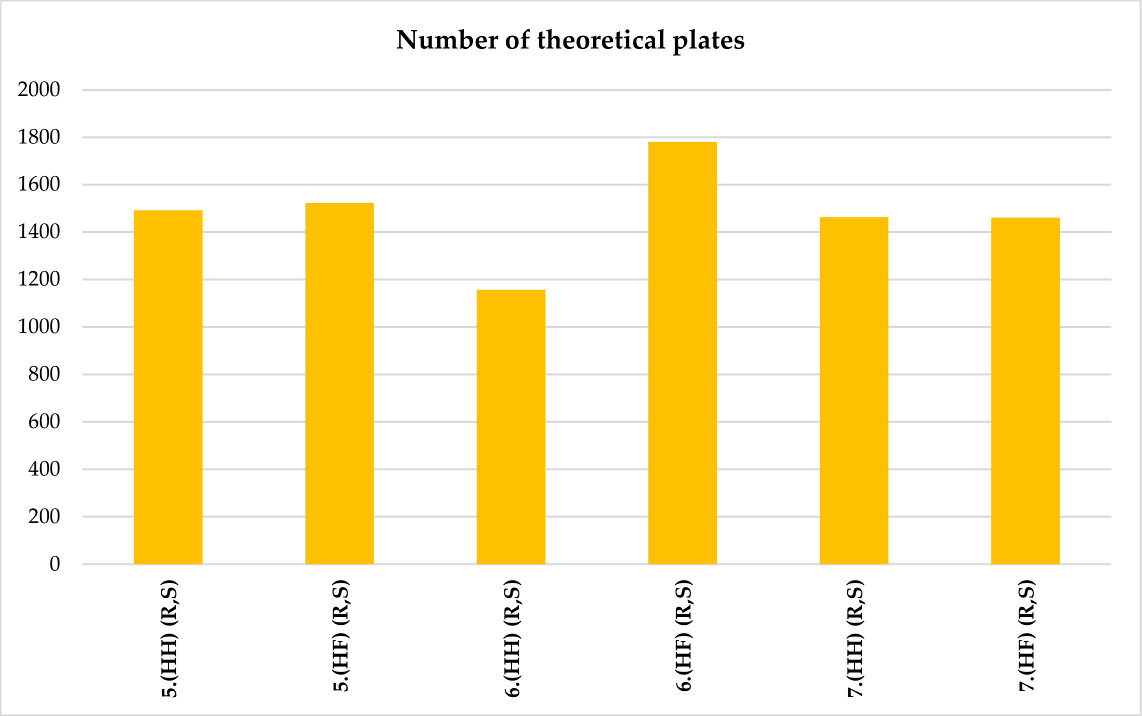

Figure S2. Theoretical plate values on the MIG column in the isocratic elution, ACN/H_2O (99:1 *v*/*v*), with a 1 mL·min^{-1} flow rate.

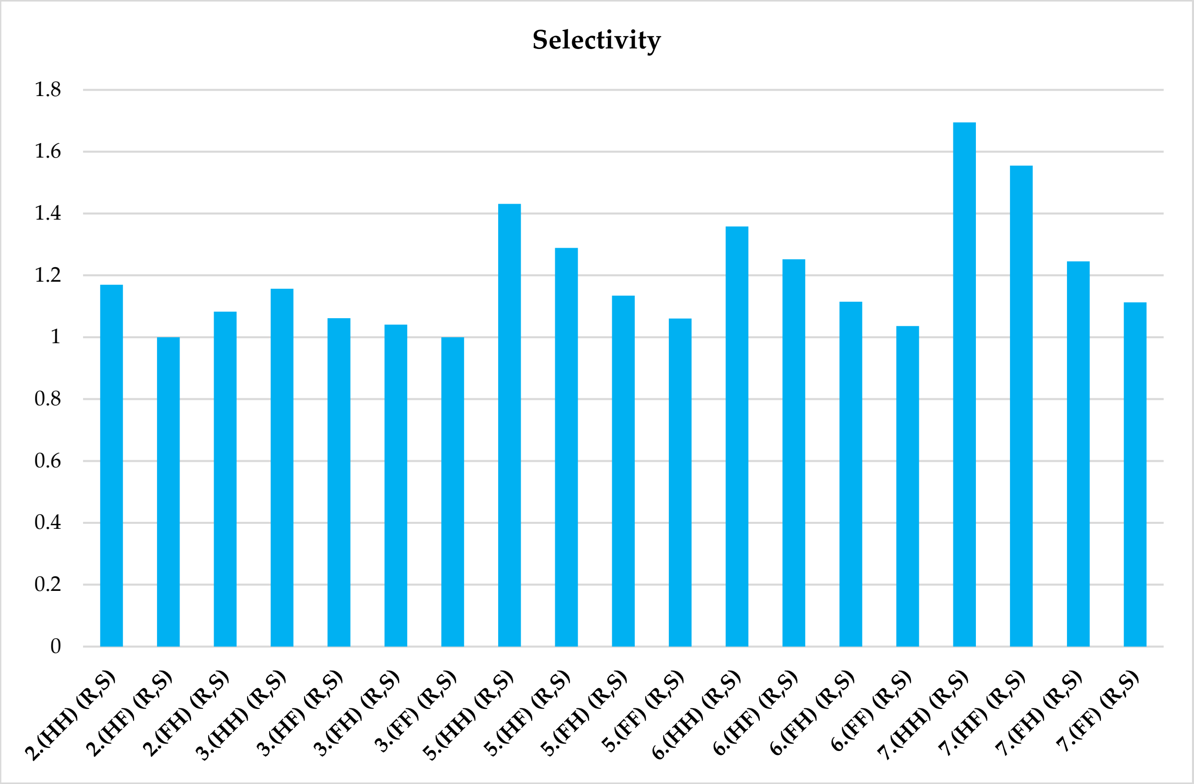

Figure S3. Selectivity values on the MIG column in the isocratic elution, ACN/H_2O (99:1 *v*/*v*), with a 1 mL·min^{-1} flow rate.

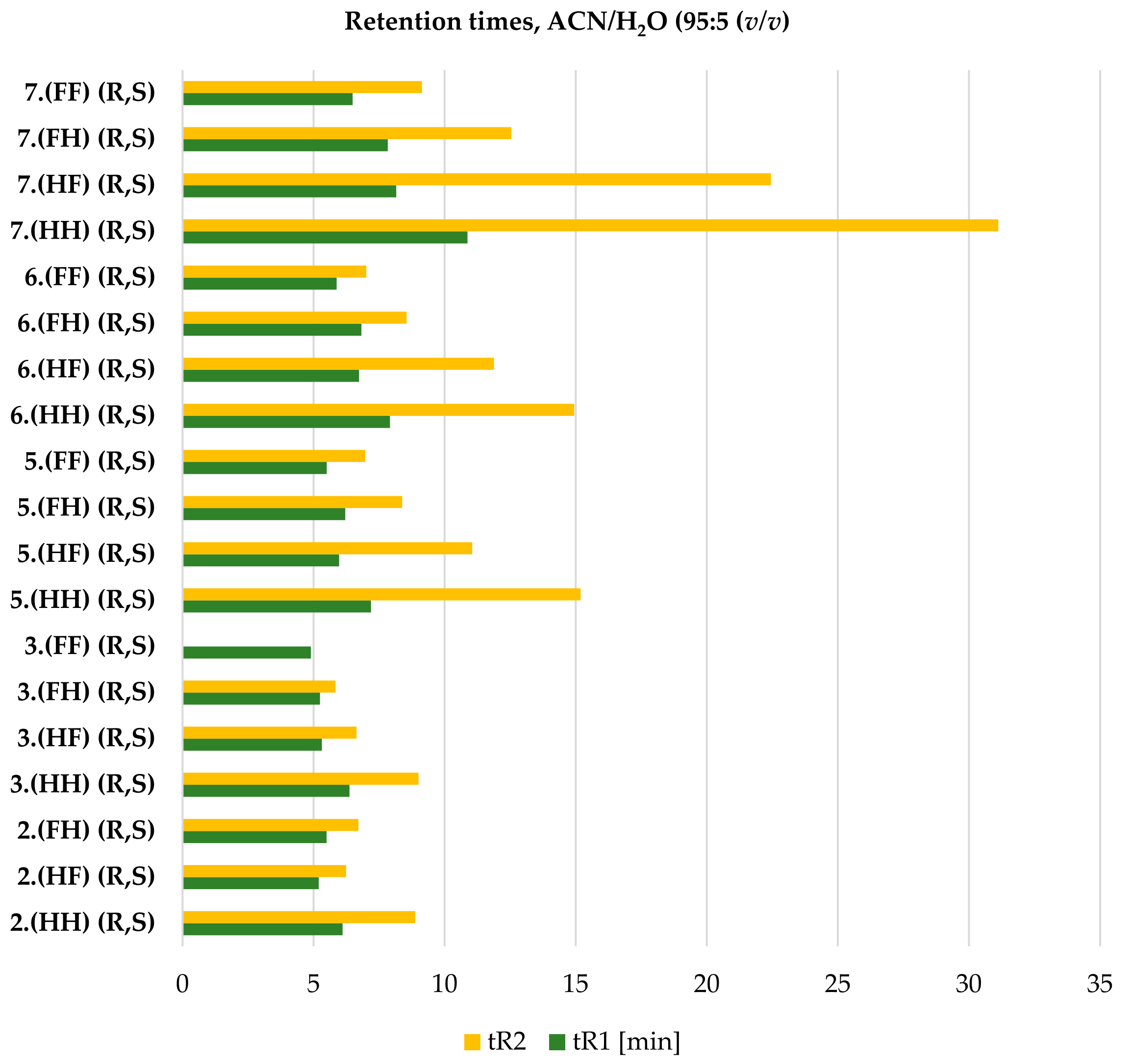

Figure S4. The retention times of the racemic mixtures on the MIG column in the isocratic elution, ACN/H_2O (95:5 *v*/*v*).

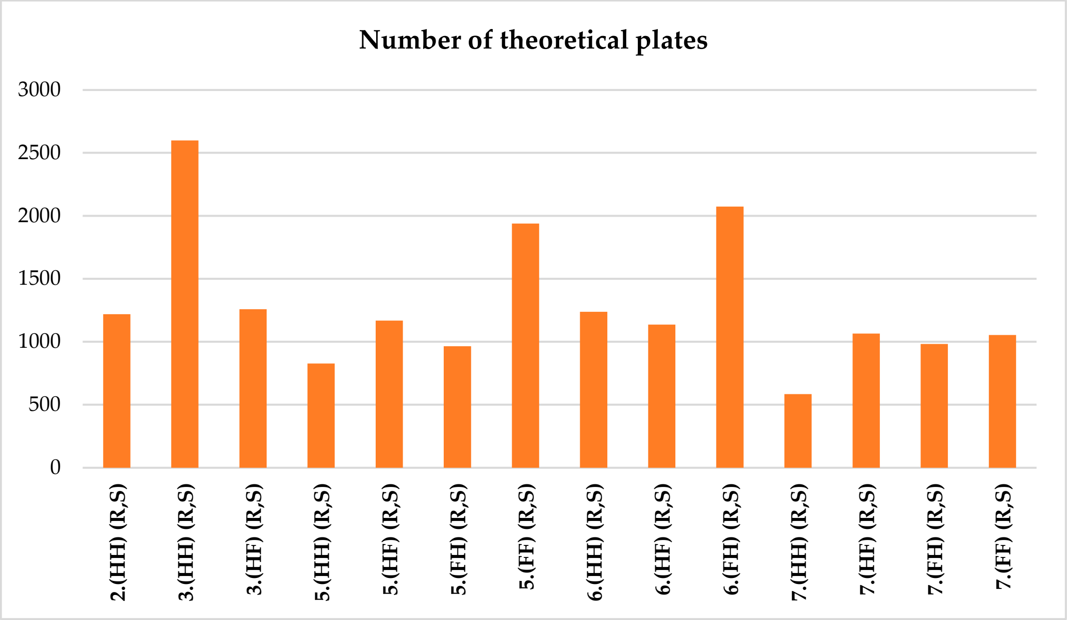

Figure S5. Theoretical plate values on the MIG column in the isocratic elution, ACN/H_2O (95:5 *v*/*v*), with a 1 mL·min^{-1} flow rate.

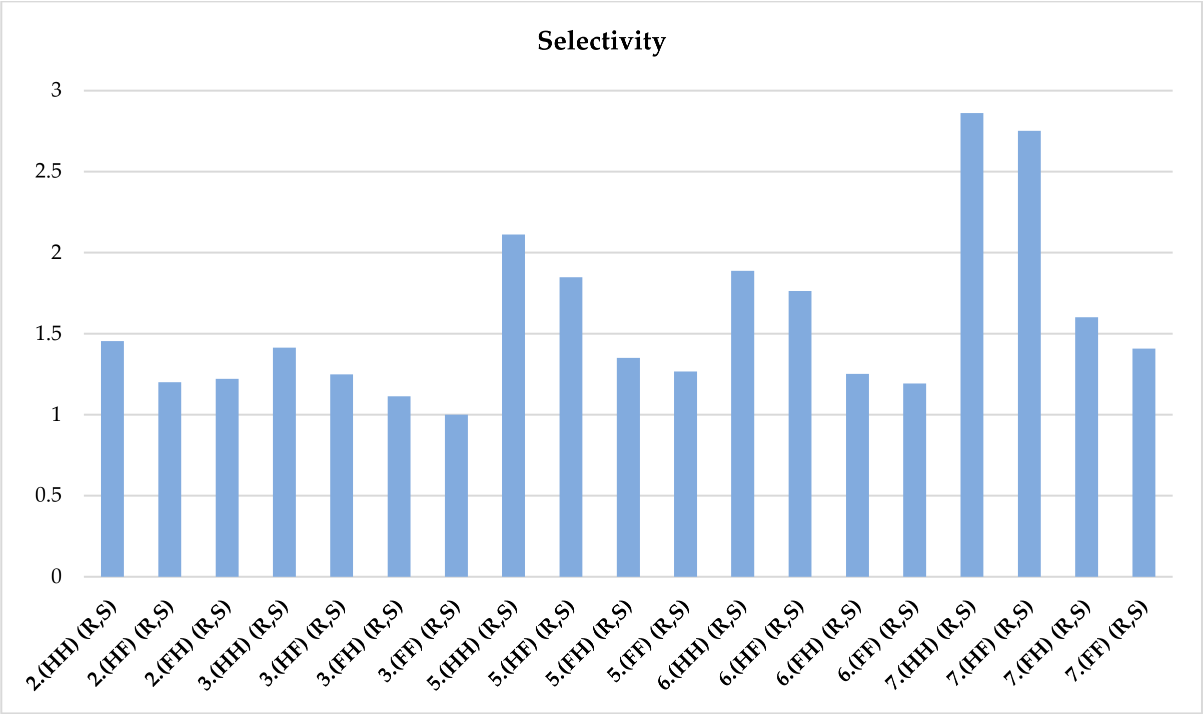

Figure S6. Selectivity values on the MIG column in the isocratic elution, ACN/H_2O (95:5 *v*/*v*), with a 1 mL·min^{-1} flow rate.

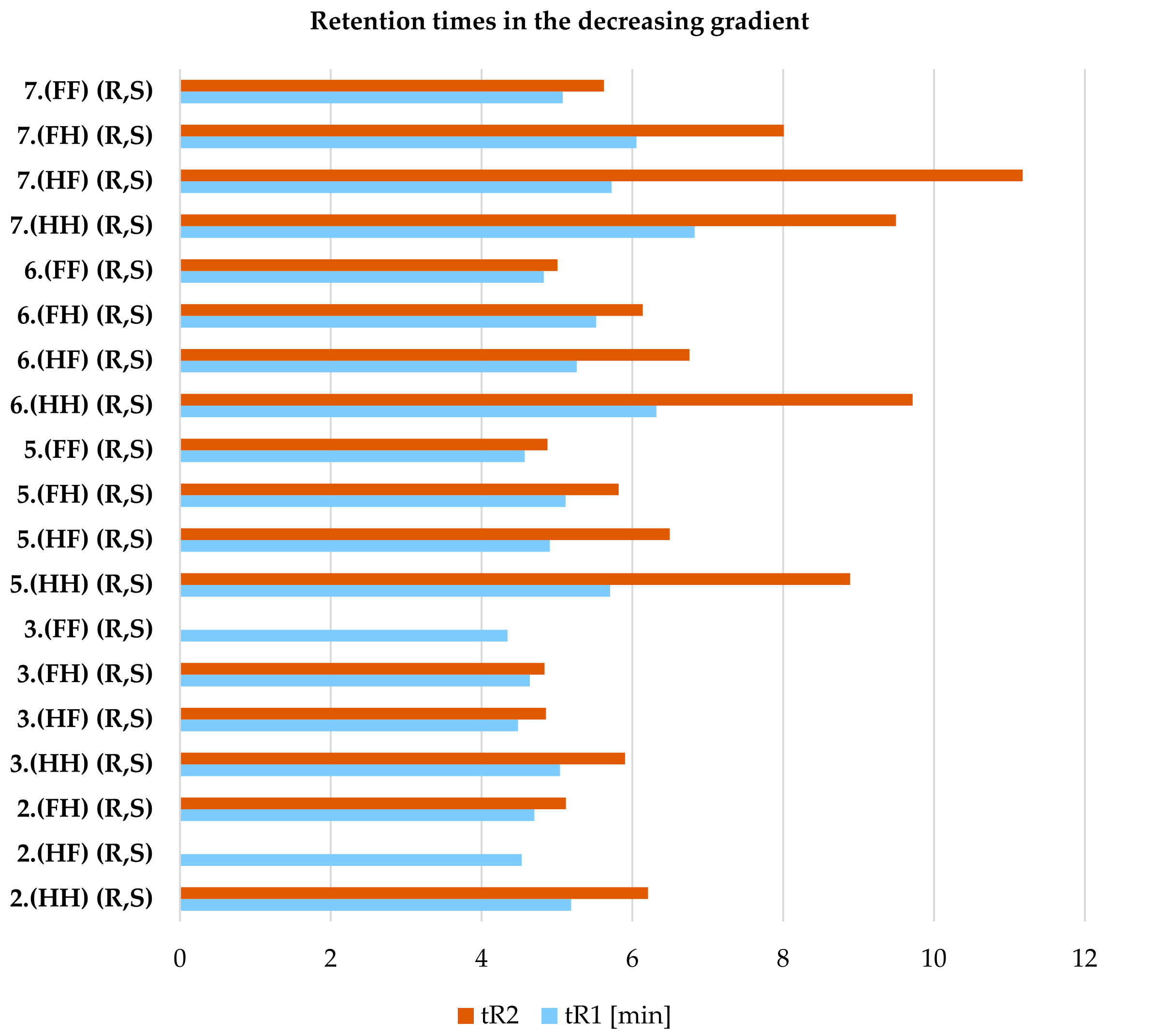

Figure S7. The retention times of the racemic mixtures on the MIG column in the decreasing gradient.

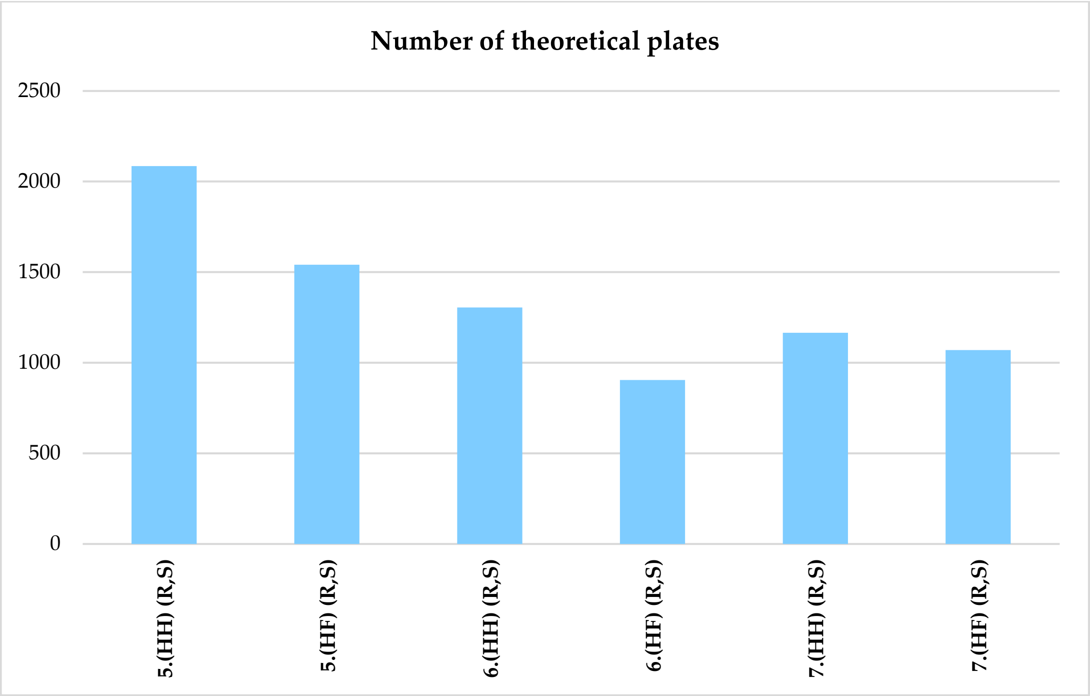

Figure S8. Theoretical plate values on the MIG column in the decreasing gradient, with a 1 mL·min^{-1} flow rate.

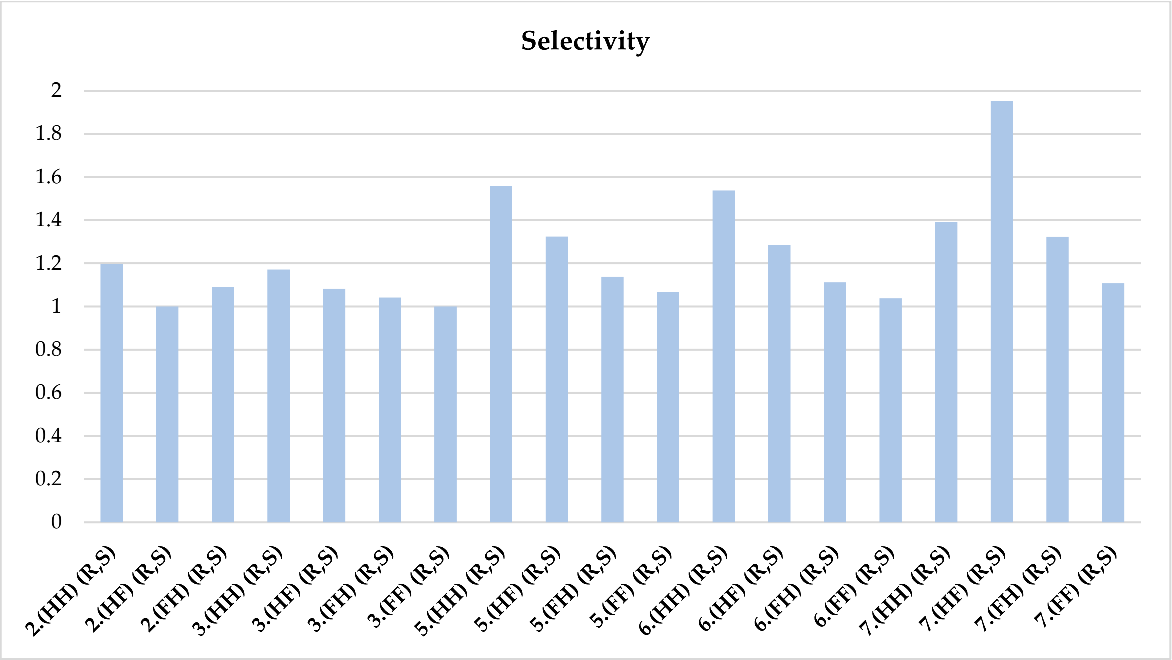

Figure S9. Selectivity values on the MIG column in the decreasing gradient, with a 1 mL·min^{-1} flow rate.

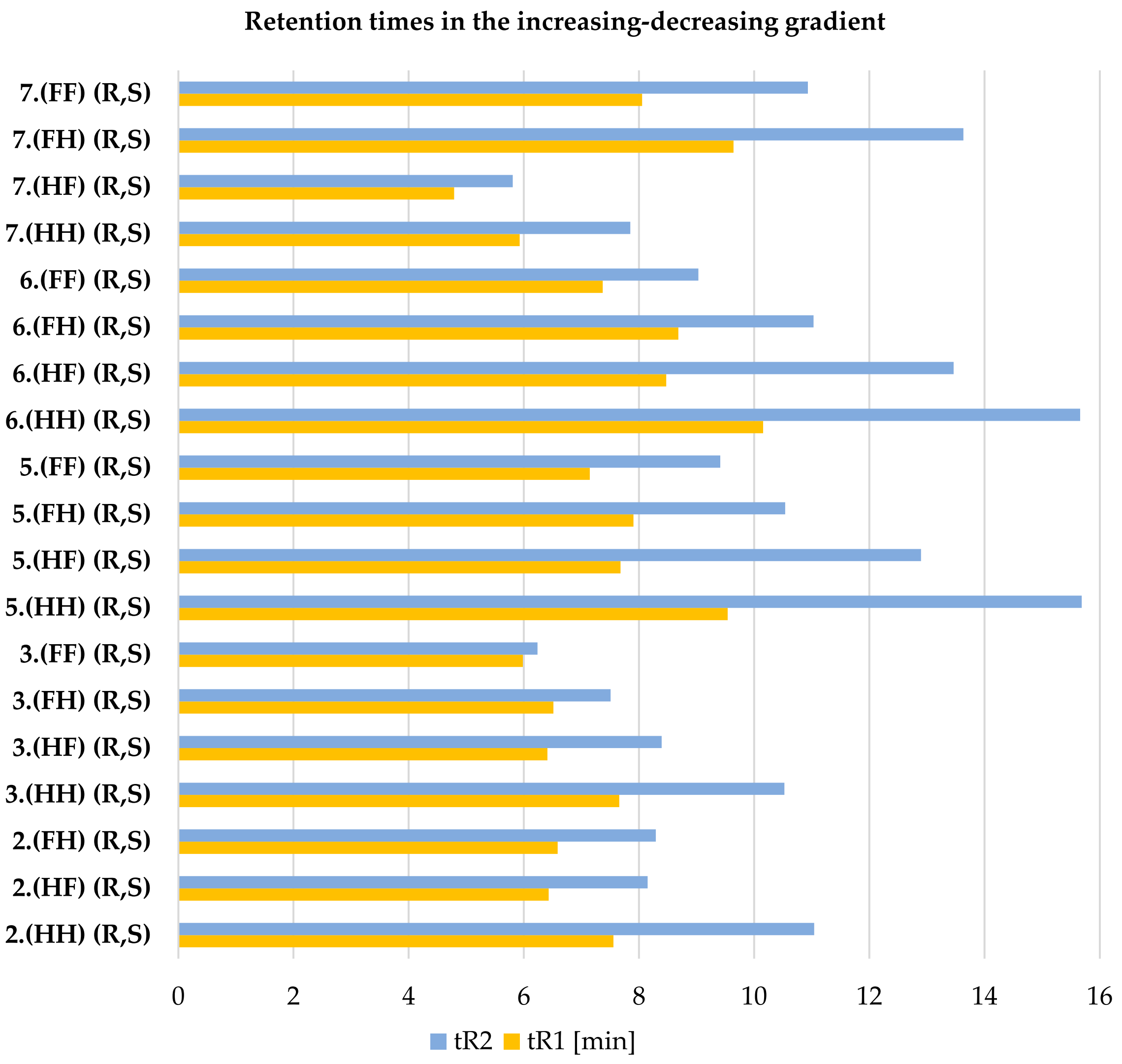

Figure S10. The retention times of the racemic mixtures on the MIG column in the increasing-decreasing gradient.

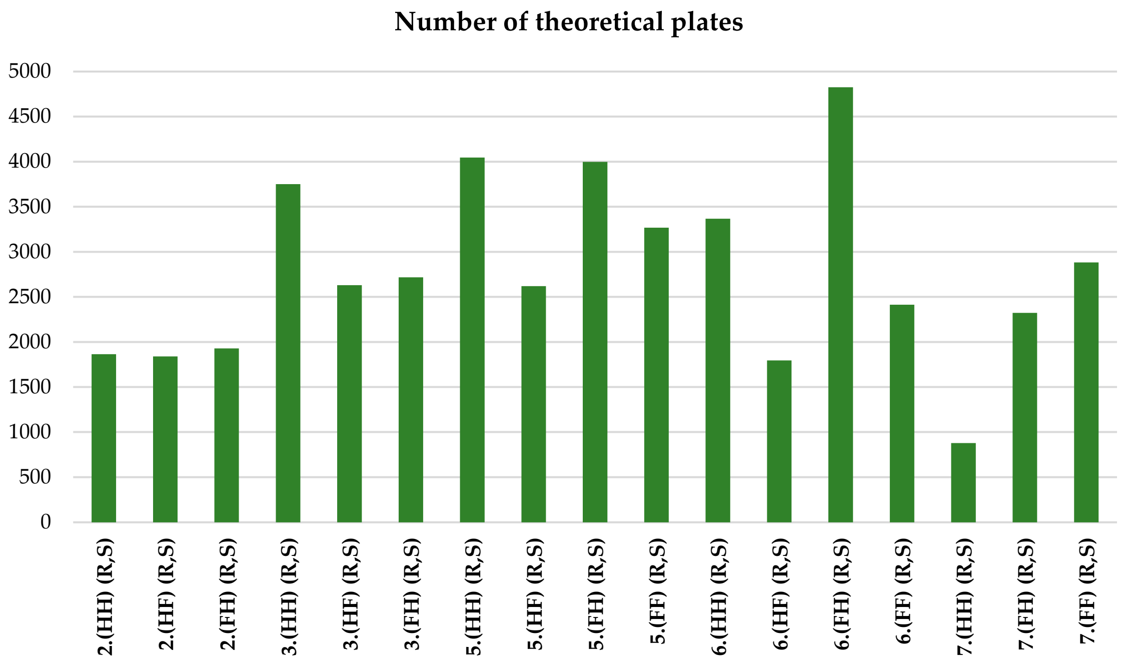

Figure S11. Theoretical plate values on the MIG column in the increasing-decreasing gradient, with a 1 mL·min^{-1} flow rate.

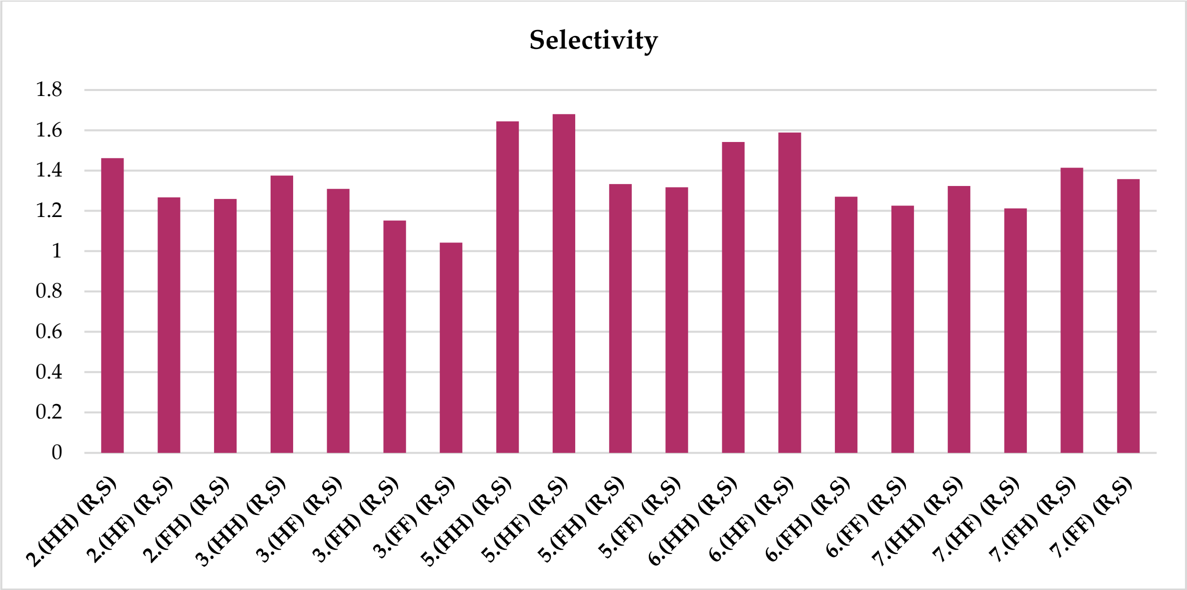

Figure S12. Selectivity values on the MIG column in the increasing-decreasing gradient, with a 1 mL·min^{-1} flow rate.

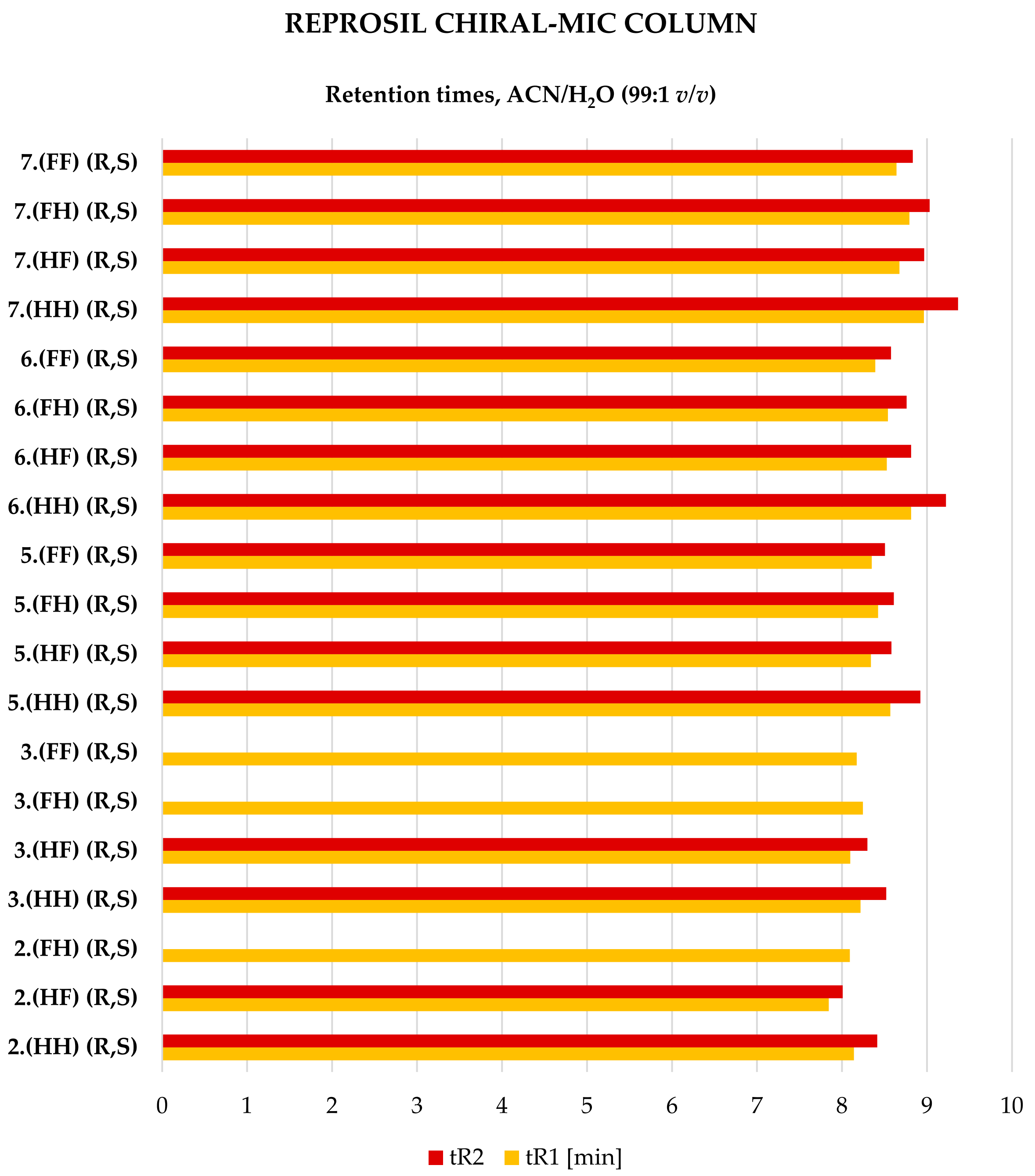

Figure S13. The retention times of the racemic mixtures on the MIC column in the isocratic elution, ACN/H_2O (99:1 *v*/*v*).

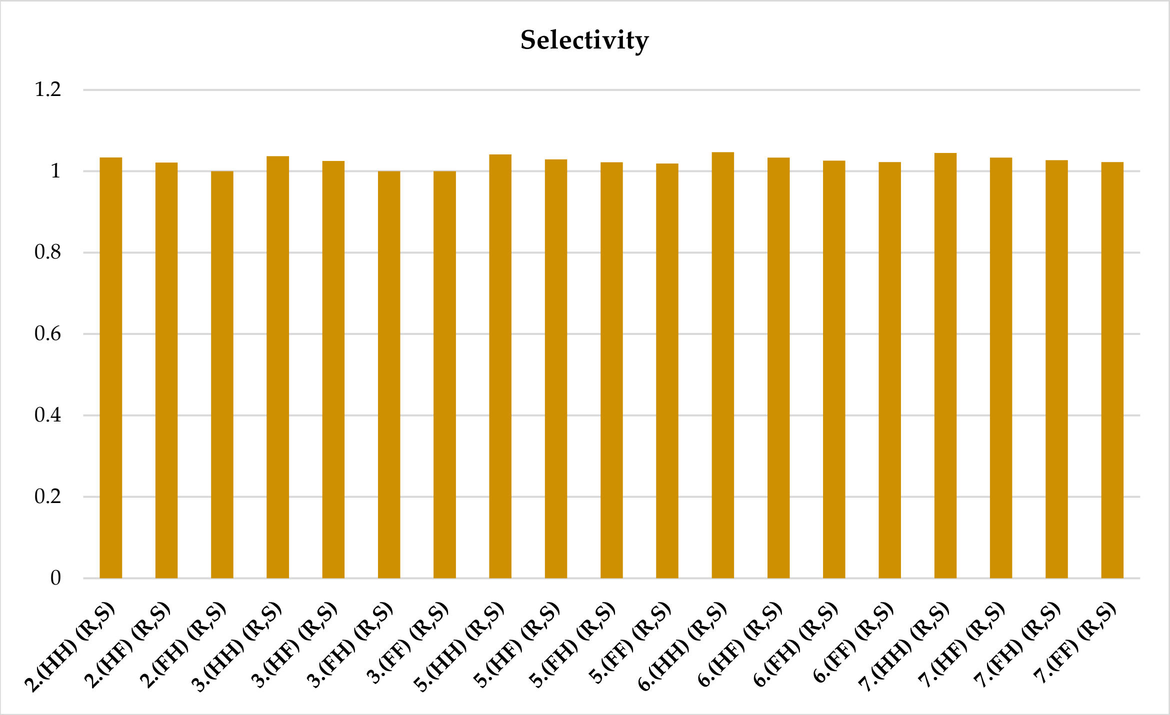

Figure S14. Selectivity values on the MIC column in the isocratic elution, ACN/H_2O (99:1 *v*/*v*), with a flow rate of 0.5 mL·min^{-1}.

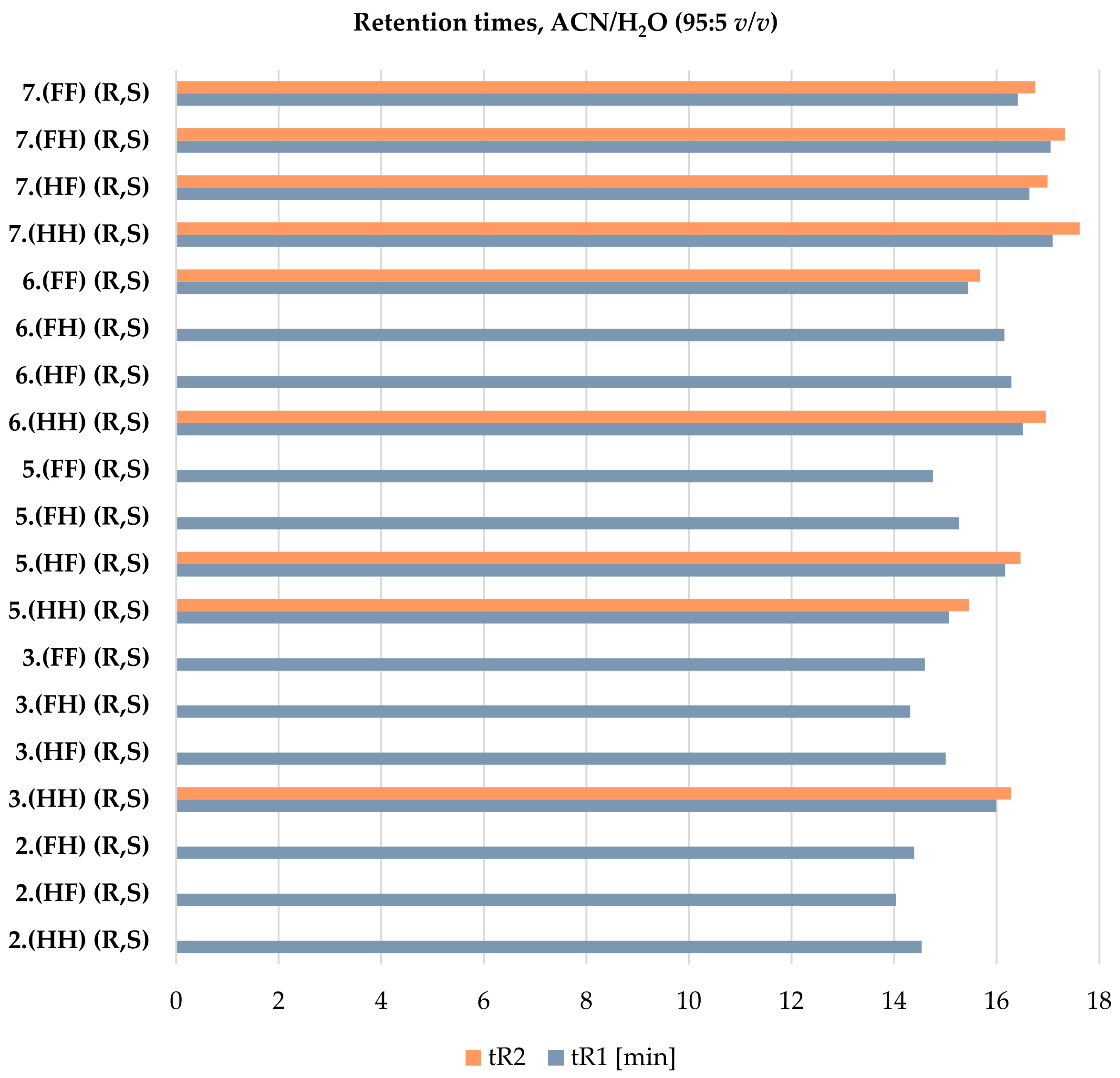

Figure S15. The retention times of the racemic mixtures on the MIC column in the isocratic elution, ACN/H_2O (95:5 *v*/*v*).

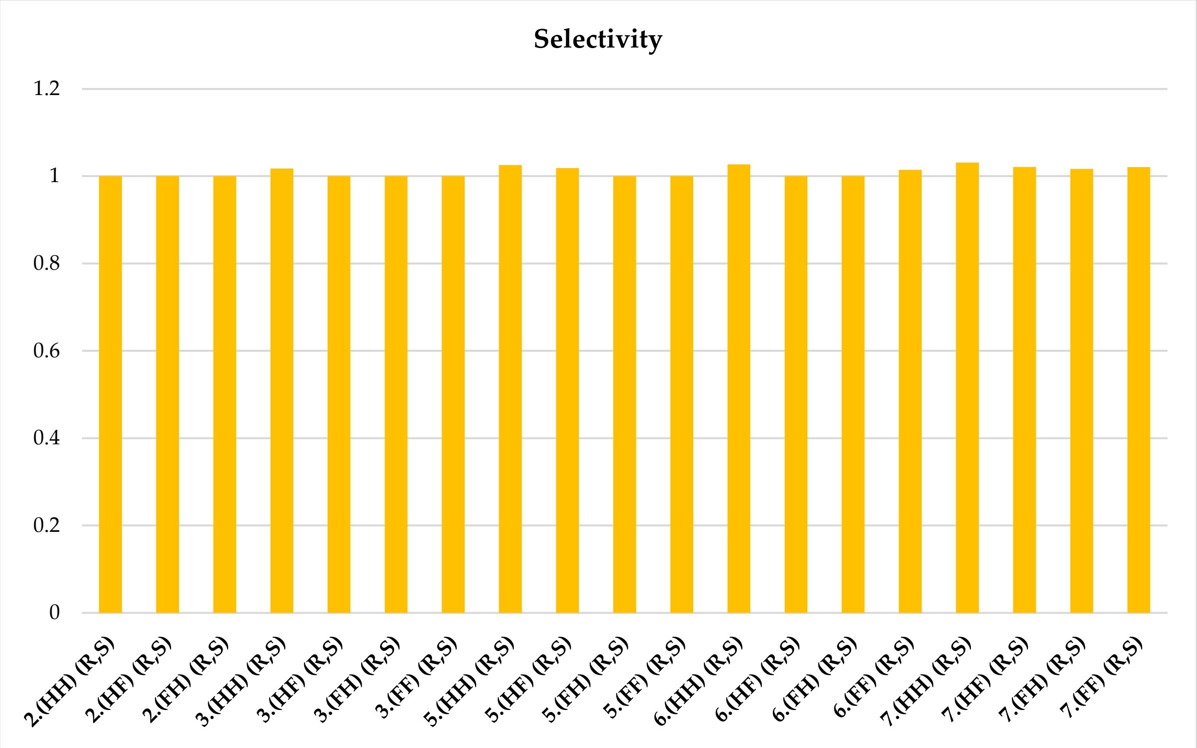

Figure S16. Selectivity values on the MIC column in the isocratic elution, ACN/H_2O (95:5 *v*/*v*), with a flow rate of 0.3 mL·min^{-1}.

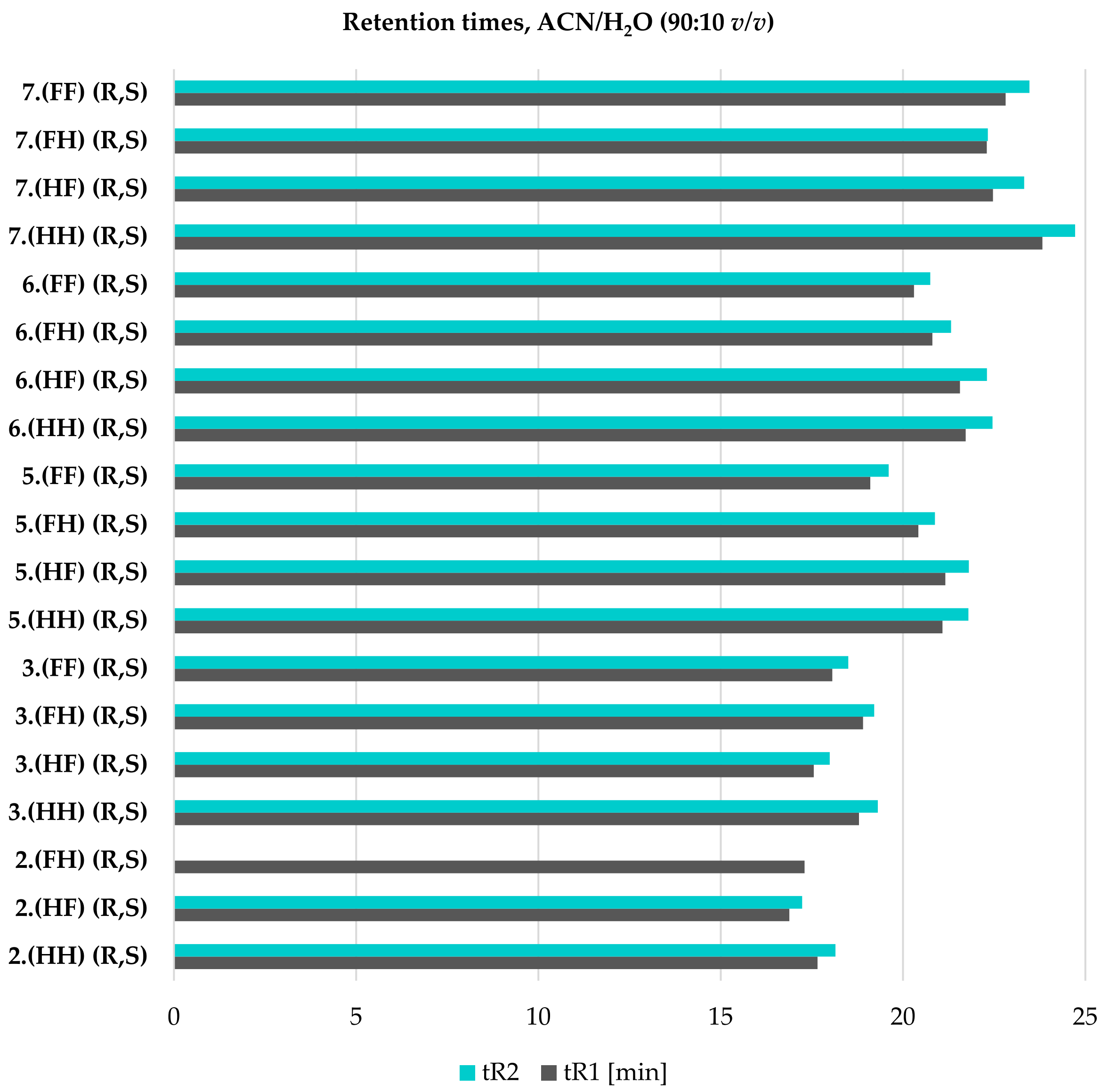

Figure S17. The retention times of the racemic mixtures on the MIC column in the isocratic elution, ACN/H_2O (90:10 *v*/*v*).

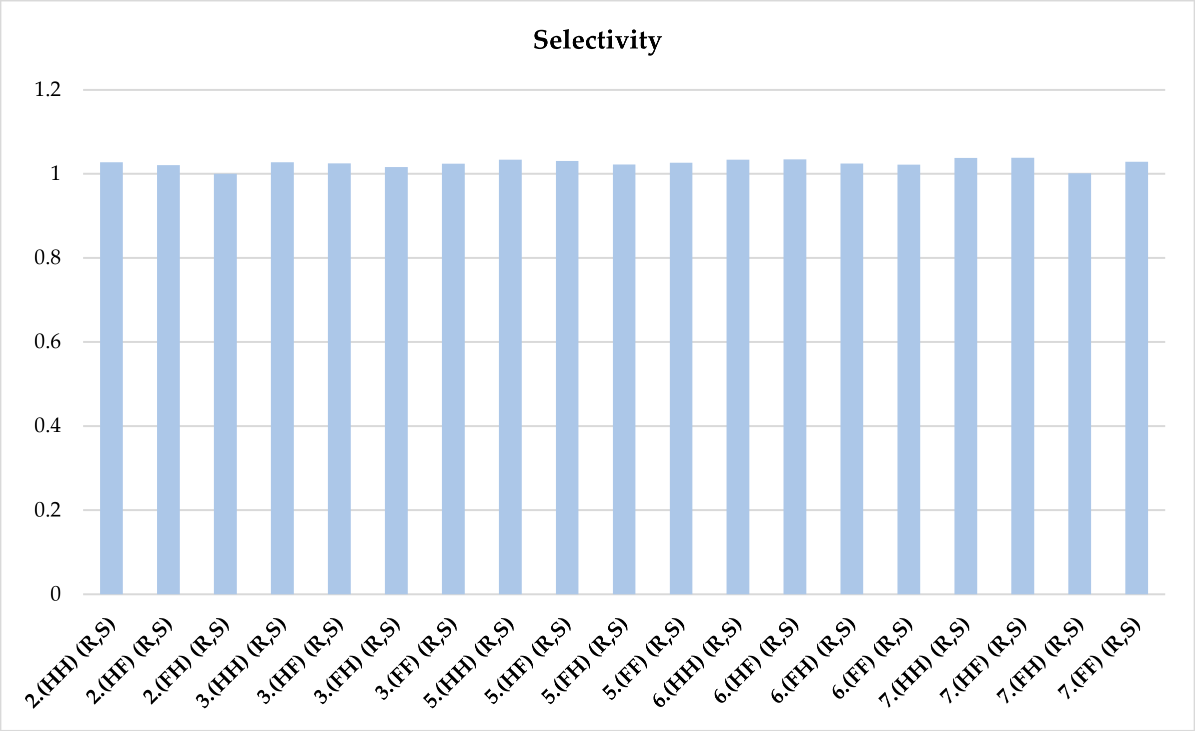

Figure S18. Selectivity values on the MIC column in the isocratic elution, ACN/H_2O (90:10 *v*/*v*), with a flow rate of 0.3 mL·min^{-1}.

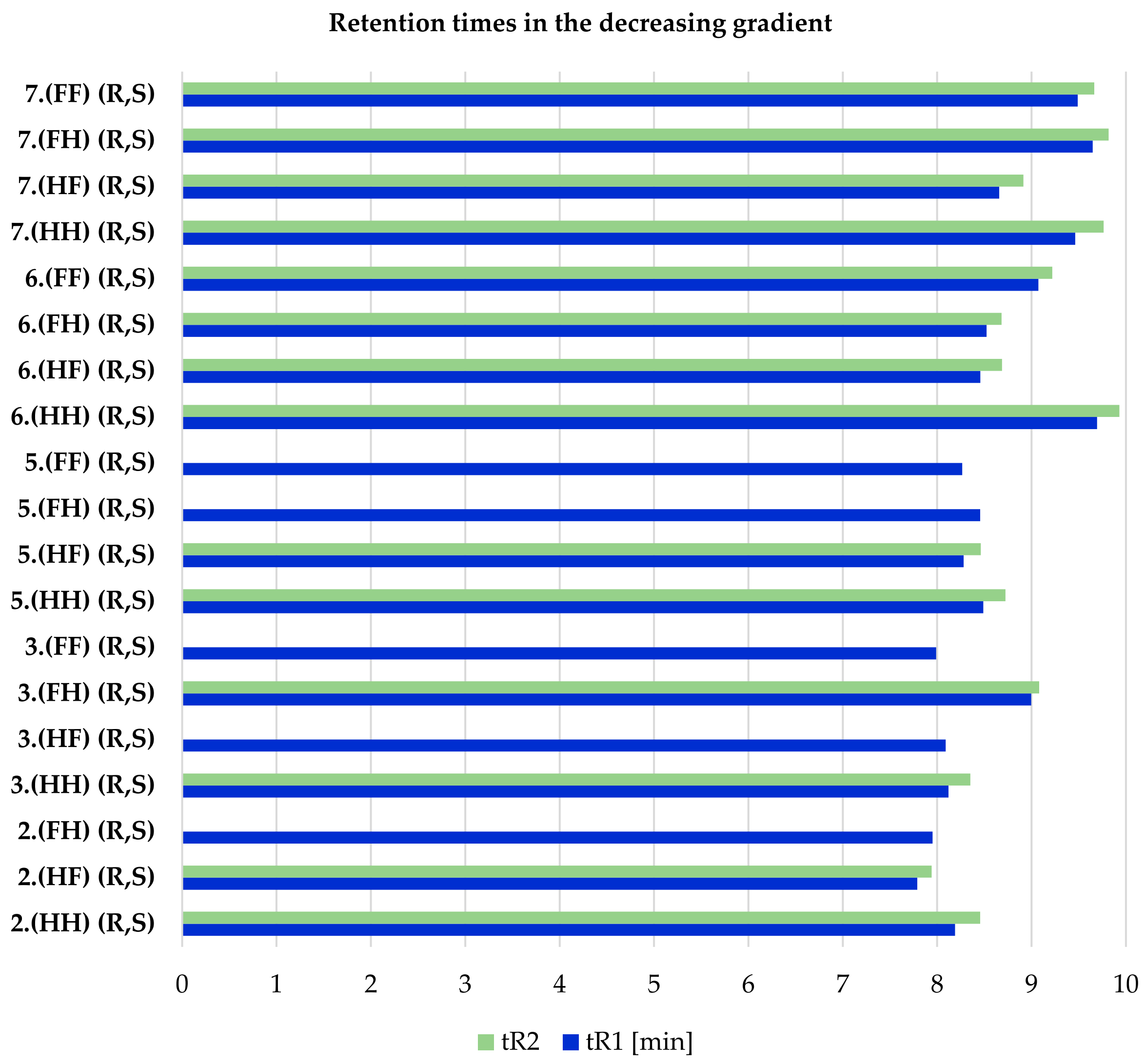

Figure S19. The retention times of the racemic mixtures on the MIC column in the decreasing gradient.

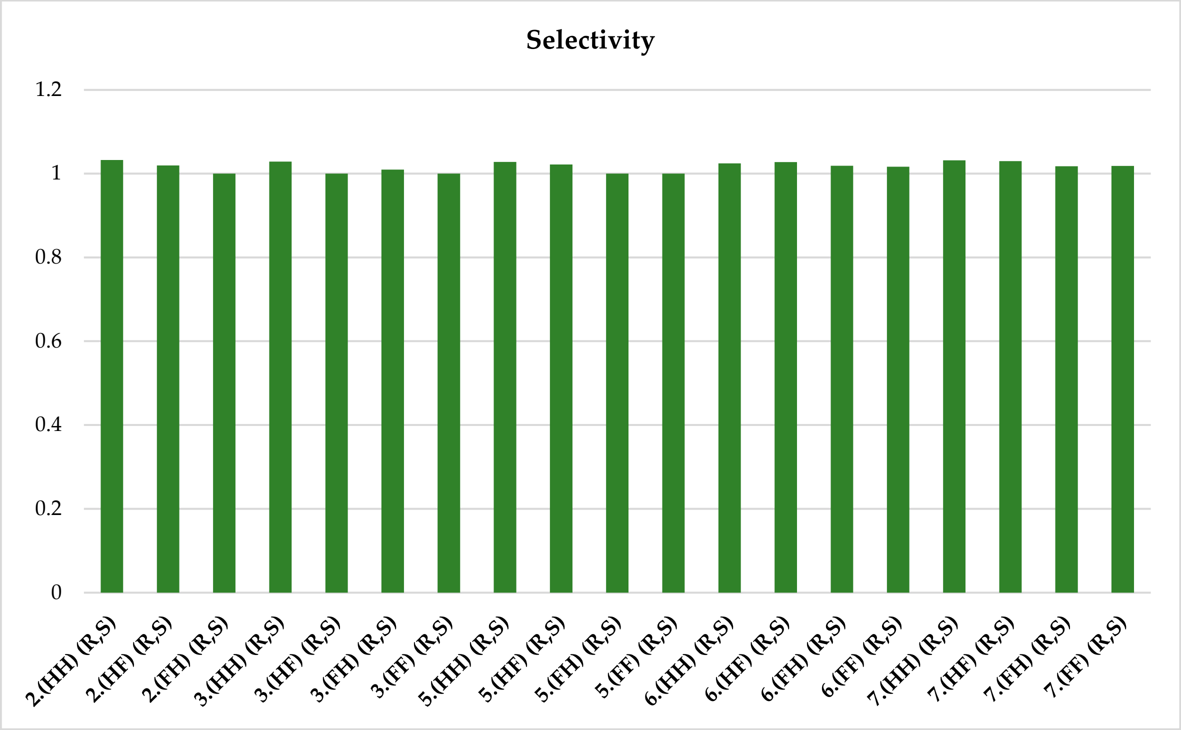

Figure S20. Selectivity values on the MIC column in the decreasing gradient, with a flow rate of 1 mL·min^{-1}.

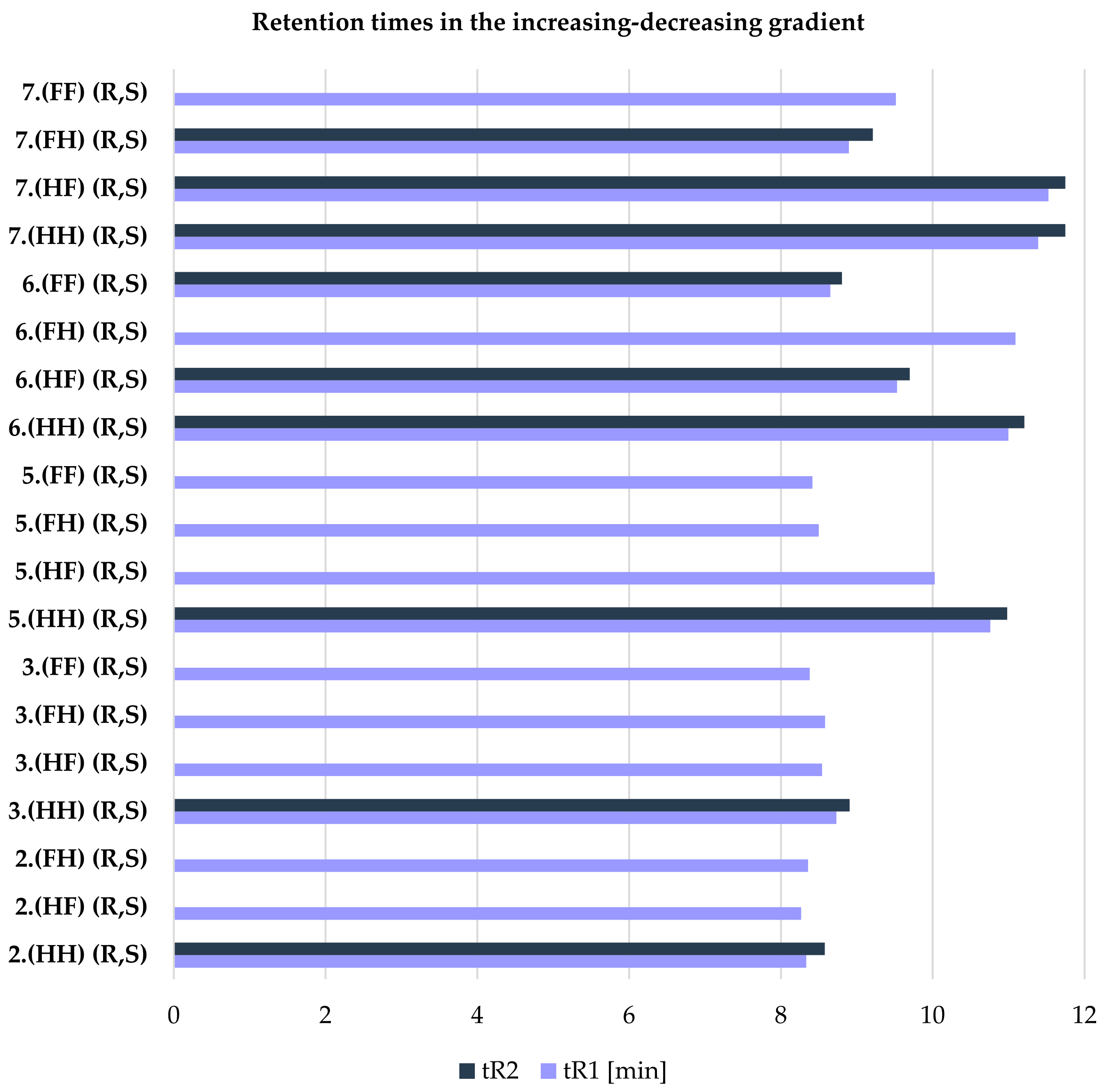

Figure S21. The retention times of the racemic mixtures on the MIC column in the increasing-decreasing gradient.

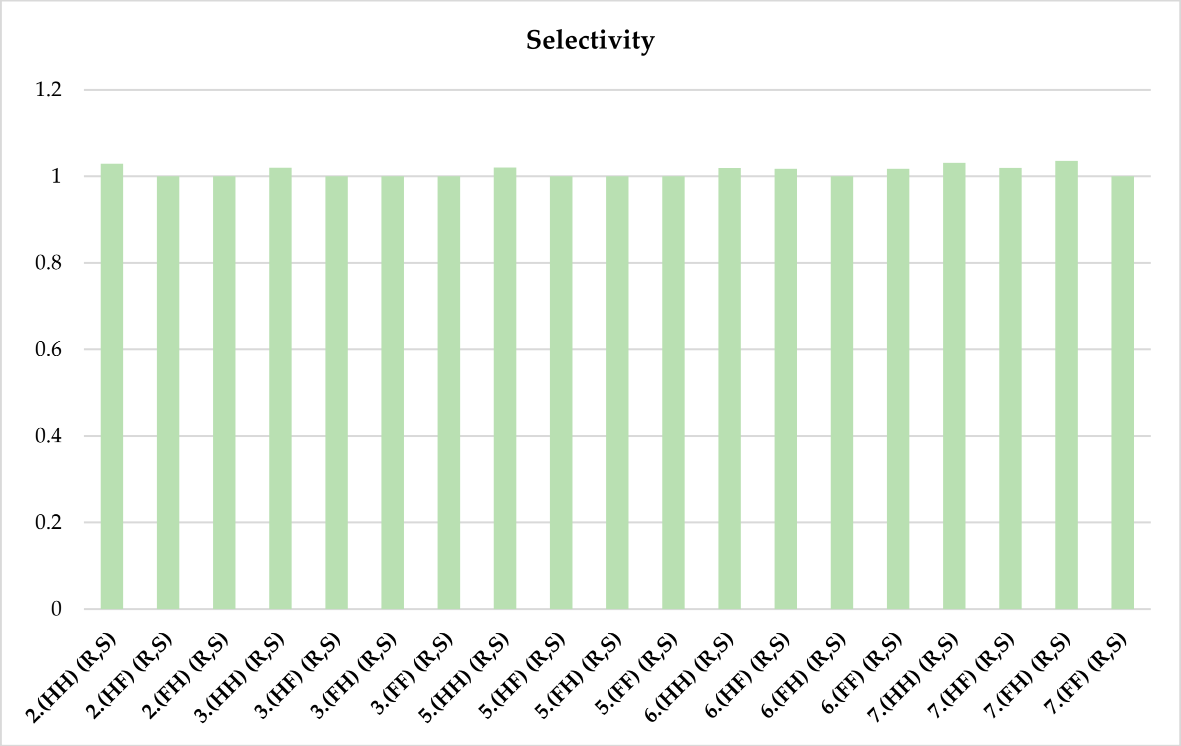

Figure S22. Selectivity values on the MIC column in the increasing-decreasing gradient, with a flow rate of 1 mL·min^{-1}.
